# HNRNPL Circularizes ARHGAP35 to Produce an Oncogenic Protein

**DOI:** 10.1002/advs.202001701

**Published:** 2021-05-01

**Authors:** Yan Li, Bing Chen, Jingjing Zhao, Qin Li, Siyuan Chen, Tianan Guo, Yuchen Li, Hongyan Lai, Zhiao Chen, Zhiqiang Meng, Weijie Guo, Xianghuo He, Shenglin Huang

**Affiliations:** ^1^ Department of Integrative Oncology Fudan University Shanghai Cancer Center Shanghai Key Laboratory of Medical Epigenetics Institutes of Biomedical Sciences Shanghai Medical College Fudan University Shanghai 200032 China; ^2^ Department of Medical Oncology Fudan University Shanghai Cancer Center Shanghai Medical College Fudan University Shanghai 200032 China; ^3^ Department of Colorectal Surgery Fudan University Shanghai Cancer Center Shanghai Medical College Fudan University Shanghai 200032 China; ^4^ Fudan University Shanghai Cancer Center and Institutes of Biomedical Sciences Shanghai Key Laboratory of Radiation Oncology Shanghai Medical College Fudan University Shanghai 200032 China

**Keywords:** ARHGAP35, cancer progression, circular RNAs, HNRNPL, oncogenic proteins, translation

## Abstract

Circular RNAs (circRNAs) are an intriguing class of widely prevalent endogenous RNAs, the vast majority of which have not been characterized functionally. Here, we identified a novel oncogenic circRNA originating from the back‐splicing of Exon2 and Exon3 of a tumor suppressor gene, ARHGAP35 (also known as P190‐A), termed as circARHGAP35. have observe that circARHGAP35 and linear ARHGAP35 have antithetical expression and functions. Interestingly, circARHGAP35 contains a 3867 nt long ORF with an m^6^A‐modified start codon and encodes a truncated protein comprising four FF domains and lacking the Rho GAP domain. Mechanistically, circARHGAP35 protein promotes cancer cell progression by interacting with TFII‐I protein in the nucleus. The RNA binding protein, HNRNPL, facilitates the formation of circARHGAP35. Clinically, circARHGAP35 is associated with poor survival in cancer patients. Our findings characterize an oncogenic circRNA and demonstrate a novel mechanism of oncogene activation in cancer by circRNA through the production of a truncated protein.

## Graphical abstract

Herein, the role of circular RNAs (circRNAs) in cancer is investigated. circARHGAP35 and linear ARHGAP35 are observed to have antithetical expression and functions in cancer. circARHGAP35 translates into an oncogenic large protein that binds to TFII‐I and promotes cancer progression, while its linear counterpart encodes a tumor suppressor. N6‐methyladenosine (m^6^A) modification of circARHGAP35 promotes its protein translation, and HNRNPL boosts the biogenesis of circARHGAP35.

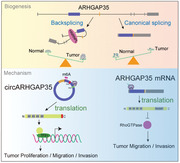

## 1 Introduction

Circular RNAs (circRNAs) comprise a large class of covalently closed RNAs produced by eukaryotic cell types and conserved among different species.^[^ ^1^, ^2^ ^]^ circRNAs are generated by a non‐canonical splicing event called backsplicing, in which the splice donor and acceptor sites are ligated to each other.^[^ ^3^ ^]^ Initially, they were considered by‐products of pre‐mRNA processing or mis‐splicing.^[^ ^4^ ^]^ In recent years, high‐throughput RNA sequencing (RNA‐seq) and bioinformatics approaches have identified thousands of circRNAs in eukaryotes.^[^ ^1^, ^5^, ^6^ ^]^ The biogenesis of circRNAs is regulated by specific cis‐acting elements and trans‐acting factors.^[^ ^7^, ^8^, ^9^ ^]^ Despite the lack of polyadenylation (poly(A)) and capping, circRNAs generally localize in the cytoplasm. Most circRNAs are derived from known protein‐coding genes and consist of one or more exons. The same genetic locus can produce both circRNAs and linear counterparts. It has been found that the expression and role of circRNAs is often not consistent with that of their corresponding linear counterparts.^[^ ^5^, ^10^, ^11^ ^]^ However, the regulation and function of circRNAs and their linear counterparts obtained from the same genetic locus are still largely unknown.

circRNAs are being increasingly recognized as promising candidates for the identification of additional layers of gene expression control. To date, despite the large number of circRNAs identified, biological functions have been investigated only for a minor fraction of circRNAs and most of these have been proposed to act as miRNA sponges.^[^ ^12^, ^13^ ^]^ Additionally, it has been demonstrated that circRNAs interact with certain proteins and act as RNA binding protein (RBP) scaffolds or decoys.^[^ ^14^, ^15^ ^]^ Intriguingly, recent studies have identified that translatable circRNAs produce previously unknown protein isoforms.^[^ ^16^, ^17^, ^18^ ^]^ Moreover, N6‐methyladenosine (m^6^A), the most abundant base involved in RNA modification, has been suggested to facilitate the efficient initiation of protein translation from circRNAs.^[^ ^19^, ^20^ ^]^ Nonetheless, the experimental data of interrogate protein‐coding circRNAs in human cancers are scarce.

Here, we identified an oncogenic circular RNA, circARHGAP35, derived from the tumor suppressor gene ARHGAP35. We show that circARHGAP35 and linear ARHGAP35 mRNA have antithetical expression and functions in hepatocellular carcinoma (HCC) and colorectal cancer (CRC). Mechanistically, circARHGAP35 contains a large ORF with an m^6^A‐modified start codon in the junction sequence and encodes a truncated protein that promotes cancer progression by interacting with the TFII‐I protein in the nucleus. We further show that production of circARHGAP35 is regulated by RBP HNRNPL.

## 2 Results

### 2.1 Identification of a circRNA Derived from the ARHGAP35 Gene

**Figure 1:**
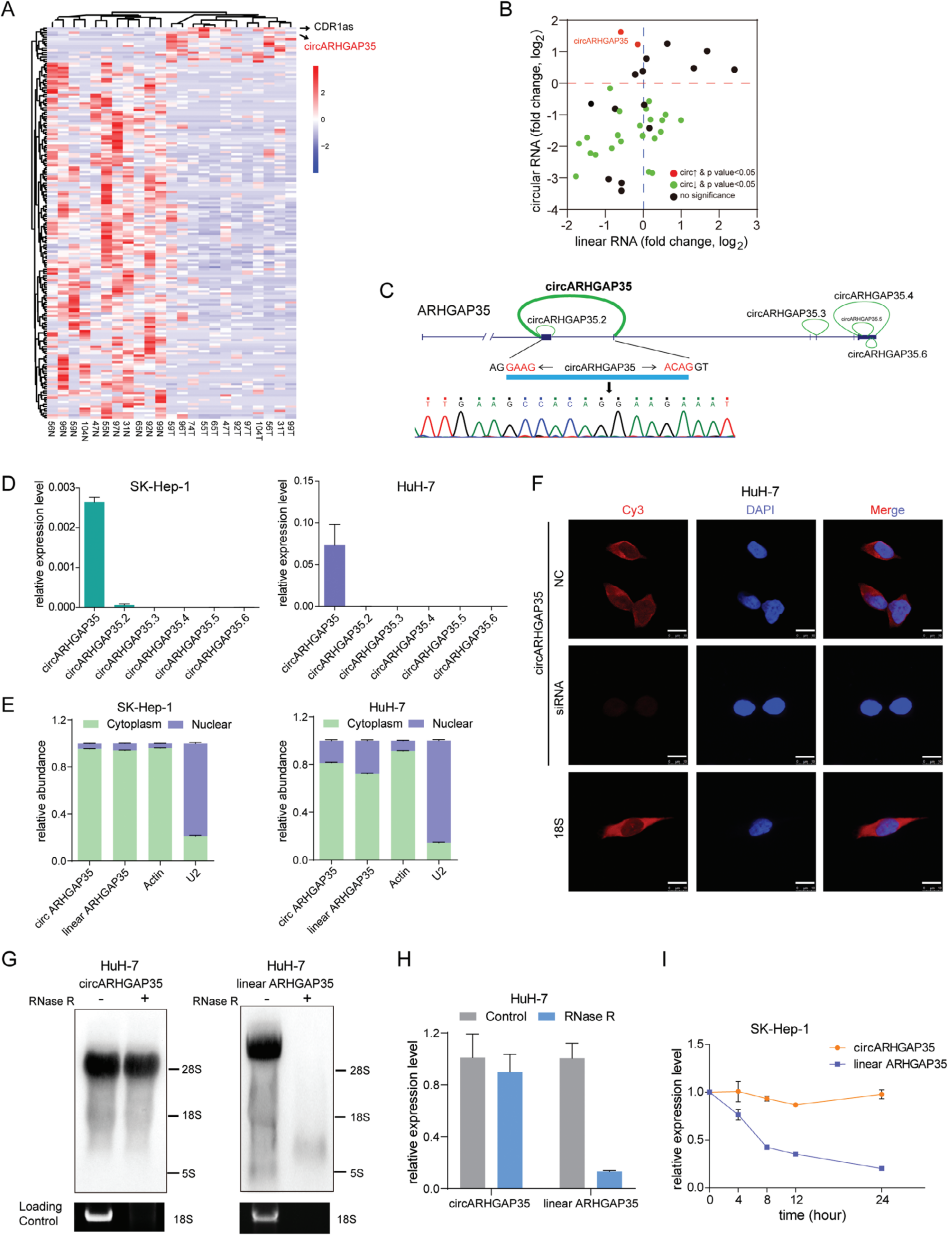
Identification of a circRNA derived from ARHGAP35 gene. A) Hierarchically clustered heatmap of circRNAs differentially expressed in 12 paired HCC and adjacent non‐cancerous liver tissue samples. Rows represent circRNAs and columns represent tissues. B) The expression of 43 circRNAs and the corresponding linear transcripts from the same gene locus detected by qRT‐PCR in 12 paired HCC and adjacent non‐tumor tissues normalized to *β*‐actin. Data were presented as the log_2_ fold change. *p* values were from paired Student's *t*‐test (*n* = 12) and adjusted with Benjamini–Hochberg method. C) The genomic loci of circular ARHGAP35 isoforms. The expression of circARHGAP35 was validated by qRT‐PCR followed by Sanger sequencing. The horizontal arrows refer to the divergent primers used to identify circARHGAP35. The junction site of circARHGAP35 is marked with vertical arrow. D) The expression of six circular ARHGAP35 isoforms in SK‐Hep‐1 and HuH‐7 cells. E) qRT‐PCR analysis of circARHGAP35 and linear ARHGAP35 RNA expression in the cytoplasm or nucleus of SK‐Hep‐1 and HuH‐7 cells. F) Identification of circARHGAP35 by fluorescence in situ hybridization (FISH) with negative control (NC) or the siRNA specifically targeting the back‐splice junction of circARHGAP35 in HuH‐7 cells. Red: circARHGAP35 probes were labeled with Cy3; Blue: nuclei were stained with DAPI. Scale bars, 10 µm. 18S was used as the cytoplasmic control. G) Northern blot for circARHGAP35 and linear ARHGAP35 without or with RNase R treatment using specific probes in HuH‐7 cells. H) qRT‐PCR analysis of circARHGAP35 and linear ARHGAP35 RNA following RNase R treatment in HuH‐7 cells. I) qRT‐PCR analysis of circARHGAP35 and linear ARHGAP35 RNA following actinomycin D treatment at the indicated time points in SK‐Hep‐1 cells. These data were represented as mean ± SEM. Results were performed in at least three independent experiments.

We profiled circRNA transcripts from 12 paired hepatocellular carcinoma (HCC) and adjacent cancer tissues using RNA‐sequencing (RNA‐seq) analyses of ribosomal RNA‐depleted total RNA. Across all samples, we annotated 42959 circRNAs with at least 2 unique backsplice reads. The majority of these circRNAs (96.0%) originated from 9799 annotated genes, which generate 1–122 circRNAs per gene. The median length of the exonic circRNAs was 509 nt. circRNA expression robustly segregated tumor cells from normal cells in the multidimensional scaling analysis (Figure S1A, Supporting Information). Differential expression analysis revealed that most of the dysregulated circRNAs (177) were downregulated in HCC, while a small portion of circRNAs (16) including CDR1as, a well‐described circRNA, were upregulated (**Figure** **1**A). Notably, we observed that most of the dysregulated circRNAs did not correlate with their linear counterpart (Figure S1B, Supporting Information). We further compared the expression levels of 43 circRNAs with those of their linear counterparts from the same gene locus in another cohort of HCC and adjacent non‐cancerous tissues using the qRT‐PCR assay (Figure 1B). Interestingly, one circRNA that backspliced Exon2 and Exon3 of the ARHGAP35 gene (hereafter referred to as circARHGAP35) was upregulated in HCC, while its linear counterpart ARHGAP35 was downregulated (Figure 1B). Our RNA‐seq data and circBase (http://www.circbase.org/) analyses indicated the presence of six putative circARHGAP35 isoforms derived from the ARHGAP35 gene locus (Figure 1C). We prioritized the investigation of circARHGAP35 since it was the most abundantly expressed form of circRNA in HCC and CRC cell lines, while other isoforms were hardly detectable (Figure 1D and Figure S1C, Supporting Information). We further validated the backsplice junction of circARHGAP35 by Sanger sequencing (Figure 1C) and qRT‐PCR analysis using outward primers (Figure S1D, Supporting Information). Additionally, we demonstrated the preferential localization of circARHGAP35 in the cytoplasm using qRT‐PCR analysis following cellular fractionation (Figure 1E and Figure S1E, Supporting Information) and fluorescence in situ hybridization with a probe spanning the back‐splicing junction (Figure 1F). Moreover, we detected the existence of endogenous circARHGAP35 by northern blot analysis using a backsplice junction‐specific probe (Figure 1G). In line with its circular nature, circARHGAP35 showed significantly greater resistance to RNase R digestion (Figure 1H and Figure S1F, Supporting Information) and a longer half‐life (Figure 1I), compared to its linear counterpart. Collectively, these results demonstrate that circARHGAP35 is a bona fide circular RNA originating from the circularization of Exon 2 and Exon 3 of ARHGAP35.

### 2.2 circARHGAP35 and Linear ARHGAP35 have Antithetical Functions in Cancer

**Figure 2:**
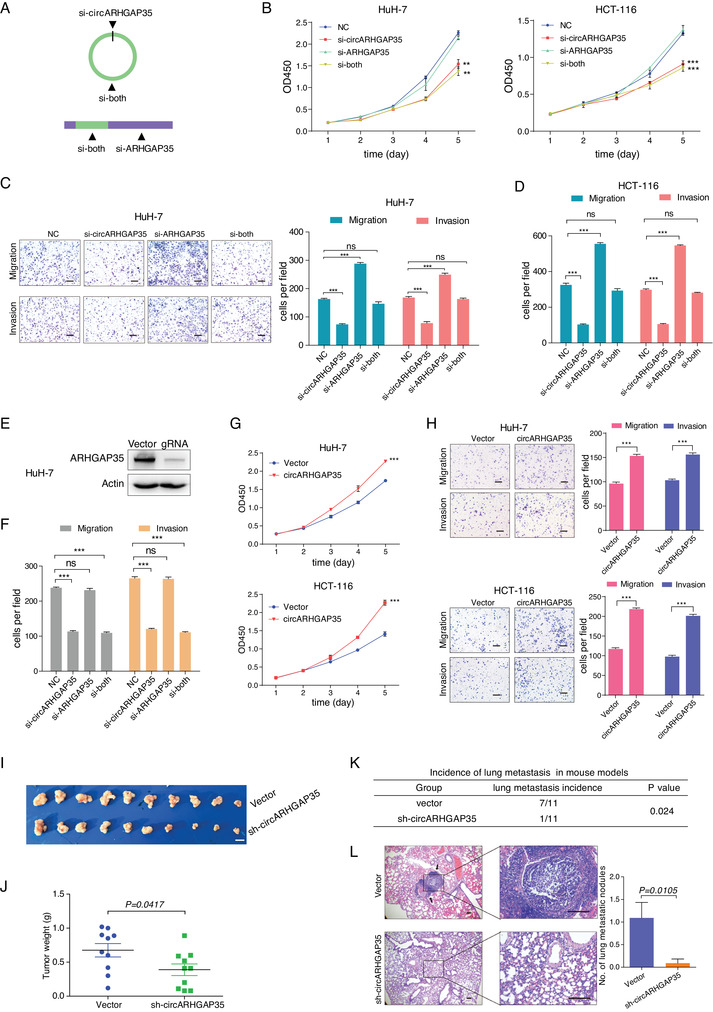
circARHGAP35 and linear ARHGAP35 have antithetical functions in cancer cell lines. A) Schematic illustration of three siRNAs specifically targeting circARHGAP35, linear ARHGAP35, and both, respectively. B) CCK‐8 proliferation assay of HuH‐7 and HCT‐116 cells transfected with the control or indicated siRNAs. C,D) Transwell migration and invasion assays of HuH‐7 (C) and HCT‐116 (D) cells performed following transfection with control or indicated siRNAs. Scale bars, 10 µm. E) Western blot validation of ARHGAP35 knockdown using CRISPR/Cas9 technology in HuH‐7 cells. F) Transwell migration and invasion assays in ARHGAP35‐knockdown HuH‐7 cells following transfection with the indicated siRNAs. G) CCK‐8 proliferation assay of HuH‐7 and HCT‐116 cells following stable overexpression of circARHGAP35. H) Transwell migration and invasion assays of HuH‐7 and HCT‐116 cells following stable overexpression of circARHGAP35. Scale bars, 10 µm. I) The effect of circARHGAP35 on tumor formation in a nude mouse xenograft model. Cells infected with either circARHGAP35 shRNA expressing lentivirus or vector control lentivirus were injected subcutaneously into the flank of each nude mouse. Scale bar, 10 mm. J) The tumor weight of the two groups. K) The effect of circARHGAP35 on tumor metastasis in a mouse tail vein injection model. Cells infected with either shRNA expressing lentivirus or vector control lentivirus were injected into the tail vein of each nude mouse (*n* = 11). Statistical analysis of the differences between the two groups was performed using the *χ*2 test. L) Hematoxylin‐eosin‐stained sections of lung metastatic nodules formed in the two groups. Slides were examined by an expert pathologist. Black arrows indicate the nodules formed in the lung. The number of metastatic nodules in the lungs of the two groups were counted and analyzed. Scale bars, 100 µm. Shown were representative images. Data were represented as mean ± SEM. Results were performed in at least three independent experiments; two‐way ANOVA and Tukey post hoc test were performed for (B) and (G); one‐way ANOVA and Dunnett post hoc test were performed for (C,D); unpaired Student's *t*‐tests were performed for (H), (J) and (L). *p < 0.05; **p < 0.01; ***p < 0.001.

To differentiate the roles of circARHGAP35 and linear ARHGAP35 in cancer, we designed three siRNAs specifically targeting the backsplice junction of circARHGAP35, its linear transcript, and both these transcripts, respectively (**Figure** **2**A). The interference efficiencies of different siRNAs were confirmed by qRT‐PCR (Figure S2A, Supporting Information). We found that circARHGAP35 depletion, but not linear ARHGAP35, significantly suppressed cell proliferation in HuH‐7, SK‐Hep‐1, and HCT‐116 cells (Figure 2B and Figure S2B, Supporting Information). In parallel, circARHGAP35 depletion remarkably reduced cell migration and invasion abilities, while an increase in cell motility was observed in the linear ARHGAP35 knockdown cells (Figure 2C,D and Figure S2C,D, Supporting Information), in concordance with previous studies.^[^ ^21^, ^22^ ^]^ Intriguingly, these effects were nullified when circular and linear ARHGAP35 were simultaneously knocked down (Figure 2C,D and Figure S2C,D, Supporting Information). To rule out the potential off‐target effects of these siRNAs, we established a linear ARHGAP35 knockdown cell line using CRISPR/Cas9 technology. In this cell line, ARHGAP35 protein level was depleted, while the expression of circARHGAP35 remained unchanged (Figure 2E and Figure S2E,F, Supporting Information). As expected, the siRNAs targeting circARHGAP35 and those targeting both isoforms reduced the migration and invasion abilities of linear ARHGAP35 knockdown cells, while the cell motility promoting effect of siRNAs targeting linear ARHGAP35 was abolished (Figure 2F). Conversely, the ectopic overexpression of circARHGAP35 increased cell proliferation, migration, and invasion (Figure 2G,H). Additionally, we designed a shRNA targeting the circARHAGP35 at the backsplice junction (Figure S3A,B, Supporting Information). Consistently we observed that circARHGAP35 shRNA treatment decreased proliferation, colony formation, migration, and invasion ability in HCC cells (Figure S3C–E, Supporting Information).

Subsequently, we sought to determine the role of circARHGAP35 in vivo. First, we tested if circARHGAP35 shRNA affected tumor growth in a nude mouse xenograft model. We observed that cells with silenced circARHGAP35 generated smaller tumors compared to controls (Figure 2I,J). Next, we evaluated the effect of circARHGAP35 on tumor metastasis in vivo. We employed a mouse tail vein injection model and performed histopathological examinations to detect lung metastasis at 10 weeks post inoculation. Remarkably, mice with circARHGAP35 silencing showed a much lower lung metastatic proportion compared to the vector control group (9% vs. 63.6%; *p* = 0.024) (Figure 2K,L). In addition, we observed that linear ARHGAP35 overexpression remarkably inhibited the tumorigenic lung metastatic ability of HCT‐116 cells (10% vs 70%; *p* = 0.02) (Figure S3F–I, Supporting Information).

Collectively, these findings suggest that circARHGAP35 and linear ARHGAP35 exert antithetical roles in tumors: circARHGAP35 promotes tumor cell growth, migration, invasion, and metastasis, while linear ARHGAP35 acts as a tumor suppressor and inhibits cell migration and invasion.

### 2.3 circARHGAP35 Encodes a Functional Protein

**Figure 3:**
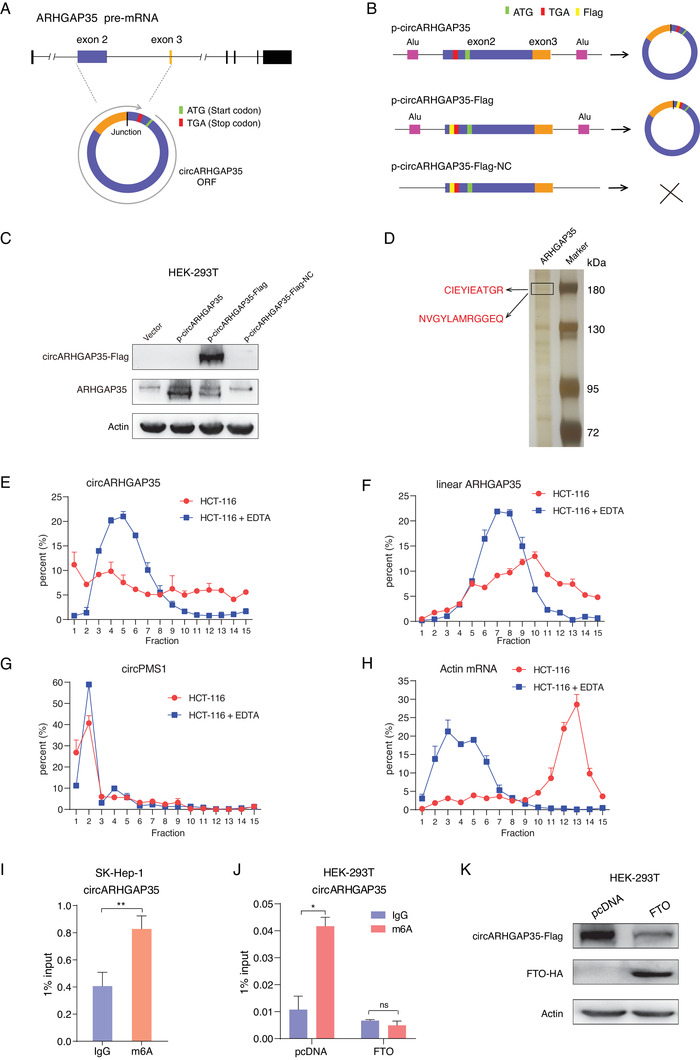
circARHGAP35 encodes a protein. A) Schematic representation of a putative open reading frame (ORF) in circARHGAP35. The junction is present inside the ORF. Start and stop codons are indicated in green and red, respectively. B) Schematic representation of the expression constructs. circARHGAP35 sequence was inserted into a circular RNA expression vector, which contains Alu elements to form the vector ‘p‐circARHGAP35’; Flag tag was added directly to upstream of the stop codon (TGA) to establish the construct ‘p‐circARHGAP35‐Flag’; the circARHGAP35‐Flag sequence was cloned to a linear vector to form a negative control vector ‘p‐circARHGAP35‐Flag‐NC’. The start codon and stop codon are shown in green and red, respectively. The Flag tag is shown in pale yellow. C) The indicated plasmids were transfected into HEK‐293T cells and potential proteins were detected using Western blot analysis. D) Immunoprecipitation assay was performed using ARHGAP35 antibody in SK‐Hep‐1 cells. The immunoprecipitated protein sample was subject to SDS‐PAGE and mass spectrometry analysis to identify specific sequences of the circARHGAP35 protein (red letters). E–H) Polysome profiling was performed using a linear 15% to 50% sucrose gradient. The polysomes of HCT‐116 cell cytoplasmic extracts without (HCT‐116) or with EDTA treatment (HCT‐116 + EDTA) were fractionated using sucrose density gradient centrifugation. Absorbance at 254 nm was measured. The relative levels of circARHGAP35 (E), linear ARHGAP35 mRNA (F), circPMS1 (G), and Actin mRNA (H) were analyzed by qRT‐PCR in gradient fractions in HCT‐116 cell lysates with or without EDTA treatment. circPMS1 and actin served as negative and positive controls, respectively. Relative distribution of each RNA in individual fraction represented as a percentage of total RNA. The sum of all fractions was considered as a total of one RNA. I) Methylated RNA immunoprecipitation (MeRIP) assay was performed using total RNA from SK‐Hep‐1 cells. Purified RNA was subsequently analyzed by qRT‐PCR. Nonspecific IgG was used as an isotype negative control. Statistical analysis was performed using unpaired Student's *t*‐test. *p < 0.05; **p < 0.01; ***p < 0.001. J) MeRIP was performed using total RNA from HEK‐293T cells ectopically expressing either vector or FTO. Purified RNA was subsequently analyzed by qRT‐PCR. Statistical analysis was performed using two‐way ANOVA and Tukey post hoc test. *p < 0.05; **p < 0.01; ***p < 0.001. K) FTO reduces circARHGAP35 translation. Protein was analyzed by Western blot using HEK‐293T cells co‐transfected with circARHGAP35 expression vector and FTO (or vector control).

We investigated the mechanism underlying the promotion of cancer cell proliferation and motility by circARHGAP35. Initially we hypothesized that circARHGAP35 interacts with certain proteins to exert its functions. To identify the proteins binding to circARHGAP35, we tagged circARHGAP35 with MS2 hairpins and co‐expressed it with MS2‐GST fusion protein (MS2 binding protein fused with GST tag) (Figure S4A, Supporting Information), and performed a pulldown assay using glutathione beads, followed by mass spectrometry. Mass spectrometry results suggested that circARHGAP35 interacted with EIF3I and EIF2S1—proteins associated with protein translation (Figure S4B, Supporting Information). Using the RNA immunoprecipitation (RIP) assay, we verified the interaction between circARHGAP35 and EIF3I (Figure S4C, Supporting Information). Moreover, the analysis of the circARHGAP35 sequence revealed the presence of a 3867 nt open reading frame (ORF), spanning from the AUG start codon of the host gene to the UGA stop codon, generated 44 nt beyond the backsplice junction (**Figure** **3**A and Table S6, Supporting Information). All these results prompted us to reason that circARHGAP35 encodes a protein.

To test the protein‐coding ability of circARHGAP35, we constructed a plasmid p‐circARHGAP35 containing the Exon2 and Exon3 of the ARHGAP35 gene and able to express circARHGAP35 RNA at high levels (Figure 3B, upper). We obtained a construct from p‐circARHGAP35 containing a Flag tag coding sequence immediately upstream of the stop codon, such that the production of the Flag‐tagged protein progressed only upon formation of a circular template (p‐circARHGAP35‐Flag; Figure 3B, middle). Another plasmid with defects in forming circRNA was used as a negative control (p‐circARHGAP35‐Flag‐NC; Figure 3B, lower). These plasmids were transfected into HEK‐293T cells and potential proteins were detected by Western blot. The results showed that p‐circARHGAP35‐Flag produced a Flag‐tagged protein (Figure 3C, upper), suggesting that circARHGAP35 was able to encode a protein. Additionally, we also used an antibody which recognized the N‐terminal sequences common to the ARHGAP35 and circARHGAP35 proteins. We found that both p‐circARHGAP35 and p‐circARHGAP35‐Flag plasmids produced a specific protein band under the ARHGAP35 protein band (Figure 3C, middle). To further validate the existence of endogenous circARHGAP35 protein, we performed an immunoprecipitation assay using an N‐terminal recognizing antibody. Using mass spectrometry, we successfully observed specific peptide fragments derived from the circARHGAP35 protein (Figure 3D and Figure S4D, Supporting Information). Collectively, these results prove that circARHGAP35 encodes a large protein with a distinct C‐terminus.

We also performed polysome profiling assay using sucrose density gradient centrifugation to directly verify whether circARHGAP35 is translated. We observed the co‐sediments of circARHGAP35 and polysomes, and after EDTA‐treatment, the distribution of circARHGAP35 altered from the heavier to the lighter polysome fractions (Figure 3E and Figure S4E,F, Supporting Information), suggesting that circARHGAP35 may be translated. The co‐sediments were also observed in ARHGAP35 and Actin mRNA, but not circPMS1 (Figure 3F–H). In addition, we found that 84.6% of linear ARHGAP35, but 55.1% of circARHGAP35 sedimentate with polysome fractions (Fractions 6–15) (Figure S4G, Supporting Information). This result indicated that the translation efficiency of circARHGAP35 is relatively low, which is consistent with previous reports.^[^ ^18^, ^19^ ^]^

N6‐methyladenosine (m^6^A) has been previously reported to promote efficient initiation of protein translation from circRNAs in human cells.^[^ ^19^ ^]^ To assess the possible m^6^A modification in circARGHGAP35, we examined the m^6^A RNA immunoprecipitation sequencing (meRIP‐seq) data around the circARGHGAP35 loci adjacent to the translation start site. We observed a significant peak and three RRACH fragments (R = G or A; H = A, C, or U) at the start codon (Figure S4H, Supporting Information) resembling the consensus motif of m^6^A modification.^[^ ^23^ ^]^ To further investigate this possibility, we performed the methylated RNA immunoprecipitation (MeRIP) assay and found that circARHGAP35 was enriched in m^6^A‐specific antibody, suggesting that circARHGAP35 possessed an m^6^A modification (Figure 3I). Moreover, co‐expression of m^6^A demethylase FTO dramatically reduced the abundance of m^6^A antibody immunoprecipitated circARHGAP35 RNA (Figure 3J) and translation of circARHGAP35 protein (Figure 3K), further confirming that circARHGAP35 contained an m^6^A modification which was important for its efficient translation.

**Figure 4:**
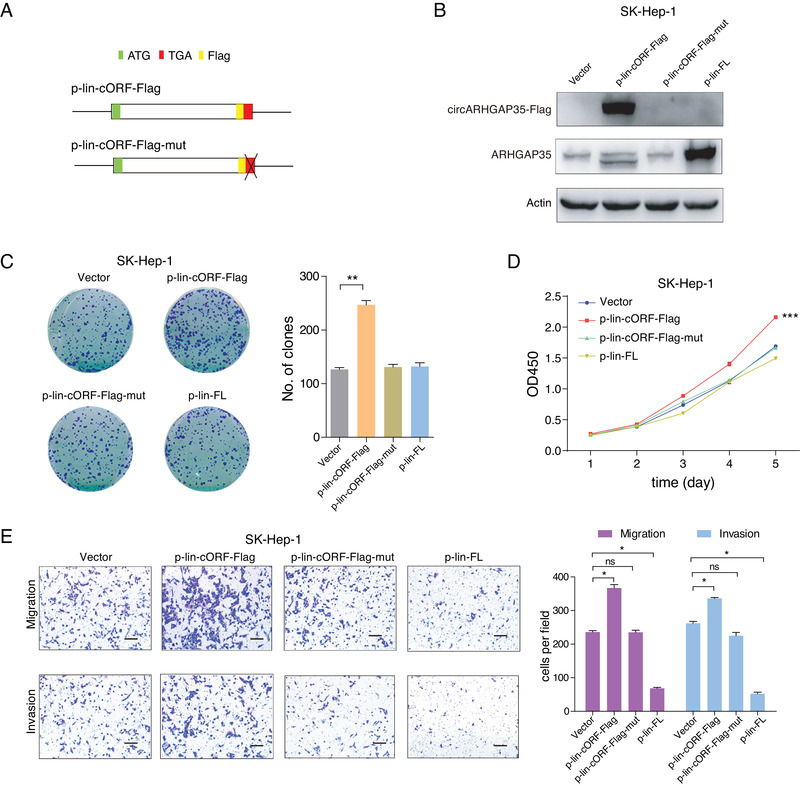
The circARHGAP35 protein has oncogenic functions. A) Schematic representation of the p‐lin‐cORF‐Flag and p‐lin‐cORF‐Flag‐mut constructs. The circARHGAP35 ORF sequence with Flag tag was inserted into a linear expression vector to form p‐lin‐cORF‐Flag. In the mutant construct, the stop codon TGA was deleted. The start codon and stop codon are shown in green and red, respectively. The Flag tag is shown in pale yellow. B) The circARHGAP35 and ARHGAP35 proteins were tested in SK‐Hep‐1 cells following the indicated lentiviral transduction. The linear ARHGAP35 full length ORF (FL) were inserted into linear vector to form p‐lin‐FL. C–E) Colony formation assay (C), CCK‐8 proliferation assay (D), and transwell migration and invasion assay (E) following the overexpression of circARHGAP35 protein, mutant protein, or ARHGAP35 protein using a linear expression vector. Scale bars, 10 µm. Data were represented as mean ± SEM. One‐way ANOVA and Dunnett post hoc test were performed for (C) and (E). Two‐way ANOVA and Tukey post hoc test were performed for (D). *p < 0.05; **p <0.01; ***p < 0.001.

To assess the biological functions of circARHGAP35 protein, an additional construct, p‐lin‐cORF‐Flag, was raised carrying the same ORF present in the circARHGAP35 but in a linear conformation (**Figure** **4**A,B). In accordance with the previous results, we found that circARHGAP35 protein drove colony formation and proliferation in cancer cell lines, while ARHGAP35 protein had little or no effect (Figure 4C,D). Furthermore, circARHGAP35 protein increased the migratory and invasive abilities of cancer cell lines, while ARHGAP35 protein played the opposite role (Figure 4E). However, the oncogenic functions of the circARHGAP35 protein were abolished upon introduction of a mutation without translated product (Figure 4C–E). To further rule out the possibility that these effects were artifacts caused by the robust overexpression of circARHGAP35 protein, we inserted the Flag‐tagged circARHGAP35 ORF sequence into the pTRIPZ vector, a Tet‐On lentiviral expression vector, and established inducible SK‐Hep‐1 cells expressing circARHGAP35 protein (Figure S5A, Supporting Information). We found that a low level of circARHGAP35 protein is sufficient to increase the proliferative, migratory, and invasive abilities of SK‐Hep‐1 cells (Figure S5B,C, Supporting Information), and this oncogenic function is similar to that of circARHGAP35 RNA (Figure S5D–G, Supporting Information). These results support the notion that circARHGAP35 functions by producing an oncogenic protein.

In summary, our findings reveal that circARHGAP35 exerts its role in cancer cell lines by translating into an oncogenic protein and that the translation of circARHGAP35 is driven by m^6^A.

### 2.4 circARHGAP35 Protein Interacts with TFII‐I in the Nucleus

**Figure 5:**
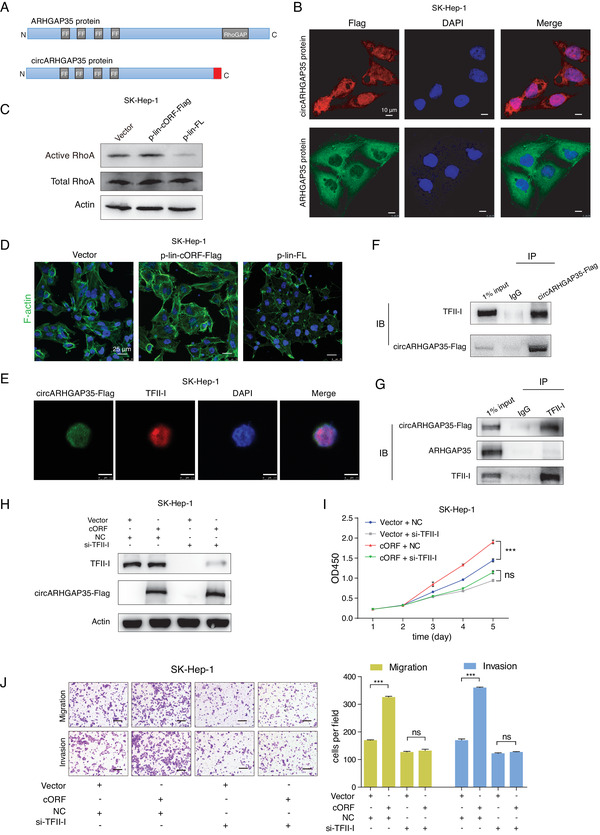
circARHGAP35 protein interacts with TFII‐I in the nucleus. A) Schematic illustrations of the protein domains of ARHGAP35 and circARHGAP35 proteins. The distinct C‐terminus of circARHGAP35 is shown in red. B) Subcellular localizations of circARHGAP35 protein and ARHGAP35 protein in SK‐Hep‐1 cells infected with circARHGAP35 expressing lentivirus or linear ARHGAP35 expressing lentivirus. circARHGAP35 protein (secondary antibody, Alexa 633, red), ARHGAP35 protein (secondary antibody, Alexa 488, green), and DAPI (blue). Scale bars, 10 µm. C) Western blot analysis of active and total RhoA in SK‐Hep‐1 cells with stable overexpression of circARHGAP35 protein, or ARHGAP35, or vector control. D) F‐actin was stained using phalloidin‐488 in SK‐Hep‐1 cells with stable overexpression of either circARHGAP35 protein or ARHGAP35 protein. Nuclei were stained with DAPI. Scale bars, 25µm. E) Immunofluorescence of circARHGAP35 protein and TFII‐I using Flag or TFII‐I antibody in SK‐Hep‐1 cells infected with circARHGAP35 expressing lentivirus. circARHGAP35 protein (secondary antibody, Alexa 488, green), TFII‐I (secondary antibody, Rhodamine, red), and DAPI (blue). Scale bars, 7.5 µm. F) Immunoprecipitation (IP) assay in SK‐Hep‐1 cells with stable overexpression of circARHGAP35 protein using either Flag or control IgG antibody, followed by immunoblotting using the TFII‐I antibody. G) IP assay in SK‐Hep‐1 cells with stable overexpression of circARHGAP35 protein using either TFII‐I or control IgG antibody, followed by immunoblotting using indicated antibodies. H) Western blot validation of circARHGAP35 protein and TFII‐I protein in circARHGAP35 protein overexpressing SK‐Hep‐1 cells following transfection with siRNA targeting TFII‐I. I,J) CCK‐8 proliferation (I) and transwell (J) assays following transfection with siRNA targeting TFII‐I in circARHGAP35 protein overexpressing SK‐Hep‐1 cells. Scale bars, 10 µm. Data were represented as mean ± SEM. Two‐way ANOVA and Tukey post hoc test were performed for (I); one‐way ANOVA and Dunnett post hoc test were performed (J), ***p < 0.001.

We explored the downstream mechanism by which circARHGAP35 contributed to tumor progression. ARHGAP35 contains four FF domains and a C‐terminal Rho GAP domain (**Figure** **5**A, upper), while the circARHGAP35 protein lacks the Rho GAP domain (Figure 5A, lower) and possesses a distinct C‐terminal instead (Figure 5A, lower, red). Surprisingly, immunofluorescence assay showed that circARHGAP35 protein was mainly found in the nucleus while ARHGAP35 protein was primarily localized in the cytoplasm (Figure 5B), indicating that they may function differently. Given the difference in the presence of the Rho GAP domain, we performed RhoA activation assay to assess the catalytic activity of circARHGAP35 and ARHGAP35 protein toward RhoA. Our results showed that the ectopic expression of ARHGAP35 protein significantly reduced RhoA activation, while circARHGAP35 protein had no significant effect on RhoA activity (Figure 5C). In concordance with the RhoA activity results, we found that the overexpression of ARHGAP35 protein significantly reduced actin‐based stress fiber formation and RhoA function in cancer cell lines, while overexpression of circARHGAP35 protein slightly increased stress fiber formation (Figure 5D). These results further confirm the antithetical roles of ARHGAP35 and circARHGAP35 in cancer.

Previous evidence suggested an interaction between the transcriptional regulator TFII‐I and the FF domains.^[^ ^24^ ^]^ We observed the nuclear localization of both TFII‐I and circARHGAP35 protein (Figure 5E), and thus we hypothesized that circARHGAP35 protein formed a complex with TFII‐I. To test this hypothesis, we performed co‐immunoprecipitation assays using anti‐Flag or anti‐TFII‐I antibody, and confirmed the interaction between TFII‐I and circARHGAP35 protein (Figure 5F,G). Conversely, the interaction between TFII‐I and ARHGAP35 was barely detectable above the background noise in HCC cells (Figure 5G). More importantly, knockdown of TFII‐I reverted the promotion of cancer proliferation, migration, and invasion by circARHGAP35 protein (Figure 5H–J). To further prove that TFII‐I transcriptional activity was regulated by circARHGAP35 protein, we explore the expression of a TFII‐I downstream target FOS, which was reported to be directly regulated by TFII‐I.^[^ ^25^ ^]^ Supporting the hypothesis, we observed that the expression level of FOS mRNA was significantly upregulated after circARHGAP35 protein stable overexpression (Figure S5H, Supporting Information). Collectively, our data reveal that circARHGAP35 exerts its oncogenic functions in cancer cell lines by partnering with TFII‐I in the nucleus.

### 2.5 HNRNPL Regulates circARHGAP35 Formation

**Figure 6:**
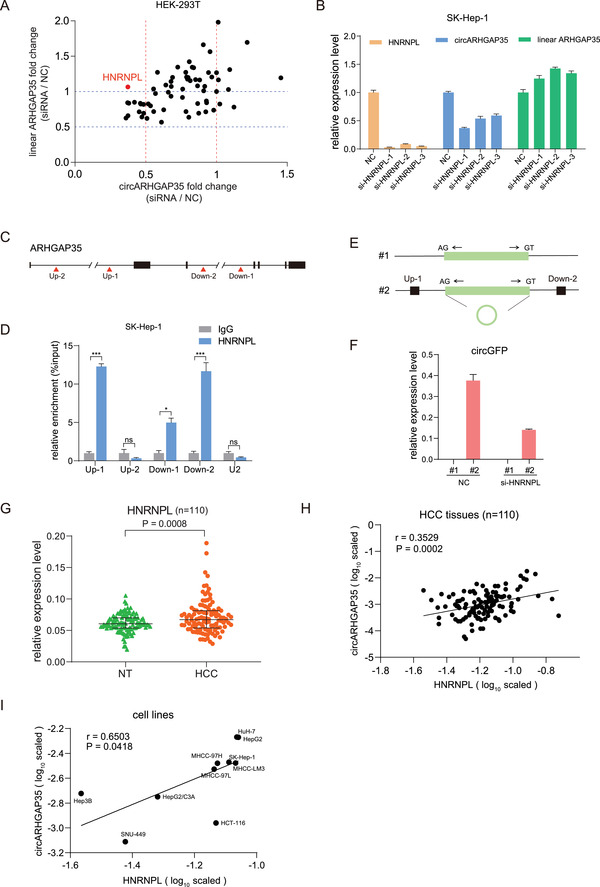
HNRNPL regulates circARHGAP35 formation. A) The expression fold change (siRNA/NC) of circARHGAP35 and linear ARHGAP35 following treatment with the siRNA library targeting 63 RBPs. The siRNA pool targeting HNRPL is highlighted in red. B) The expression of HNRNPL, circARHGAP35, and linear circARHGAP35 in SK‐Hep‐1 cells transfected with three siRNAs targeting HNRNPL. Data represent the mean ± SEM. One‐way ANOVA and Dunnett post hoc test were performed. *p < 0.05; **p < 0.01; ***p < 0.001. C) A schematic illustration of putative binding sites of HNRNPL upstream and downstream of the circARHGAP35 genomic site. D) RNA immunoprecipitation (RIP) was performed in SK‐Hep‐1 cells. qRT‐PCR was performed to quantify the RIP enriched RNA. U2 was used as a negative control. *p* Values were from unpaired Student's *t*‐tests. *p < 0.05; **p < 0.01; ***p < 0.001. E) Schematic diagram of circGFP expression vectors without or with HNRNPL binding sites (Up‐1 and Down‐2) in upstream and downstream of the circARHGAP35 genome location (#1, #2). F) qRT‐PCR analysis of the expression of circGFP using specific primer in HEK‐293T cells transfected with the indicated constructs, with siRNA targeting HNRNPL (or negative control, NC). G) The expression of HNRNPL in 110 paired HCC and adjacent non‐tumor (NT) liver tissues. Data were analyzed by paired Student's *t*‐test. H) Correlation between circARHGAP35 and HNRNPL in 110 HCC samples was determined by qRT‐PCR with *β*‐actin serving as an internal control. Statistical analysis was performed with Pearson's correlation analysis. I) Correlation between circARHGAP35 and HNRNPL in cancer cell lines. Statistical analysis was performed with Pearson's correlation analysis.

We sought to further define the molecular mechanisms driving circARHGAP35 up‐regulation in cancers. As observed in the biogenesis of other circular RNAs,^[^ ^8^, ^9^, ^26^ ^]^ we reasoned that RBPs facilitate the backsplicing of circARHGAP35. We performed a screening by using siRNAs to individually knockdown 63 RBPs known to participate in RNA splicing (**Figure** **6**A and Table S5, Supporting Information). We observed that the silencing of HNRNPL resulted in the down‐regulation of circARHGAP35 but not linear ARHGAP35 (Figure 5A). The validity of the regulation of circARHGAP35 biogenesis by HNRNPL was reinforced by individually knocking down HNRNPL using three siRNAs (Figure 6B). Previous reports suggest that HNRNPL affects circRNA biogenesis, and that it recognizes CA‐rich elements.^[^ ^27^, ^28^ ^]^ Examination of publicly available enhanced crosslinking and immunoprecipitation (eCLIP) data^[^ ^29^ ^]^ revealed that the HNRNPL binding sites flanked the circARHGAP35 locus (Figure S6A, Supporting Information). While confirming these interactions by RIP‐qPCR experiment, we observed significant enrichment of ARHGAP35 in HNRNPL, particularly at two sites situated within Intron1 and Intron3, respectively (Figure 6C,D). We further made a construct with a linear exon flanking the two binding sites (Figure 6E). We found that the circularized region could be detected by qRT‐PCR assay using divergent primers after transfection of the construct with the two HNRNPL binding sites (Figure 6F). And the detecting signal significantly decreased after co‐transfection with siRNA targeting to HNRNPL (Figure 6F). Moreover, we observed HNRNPL was significantly upregulated in HCC compared to matched non‐tumor (NT) liver tissues (Figure 6G), and is positively correlated with circARHGAP35 but not linear ARHGAP35 in HCC tissues and cells (Figure 6H,I, and Figure S6 B,C, Supporting Information). In summary, these results indicate that HNRNPL promotes the biogenesis of circARHGAP35 and its high expression levels lead to the up‐regulation of circARHGAP35 in HCC.

### 2.6 The Upregulation of circARHGAP35 is Associated with Poor Survival in Cancer Patients

**Figure 7:**
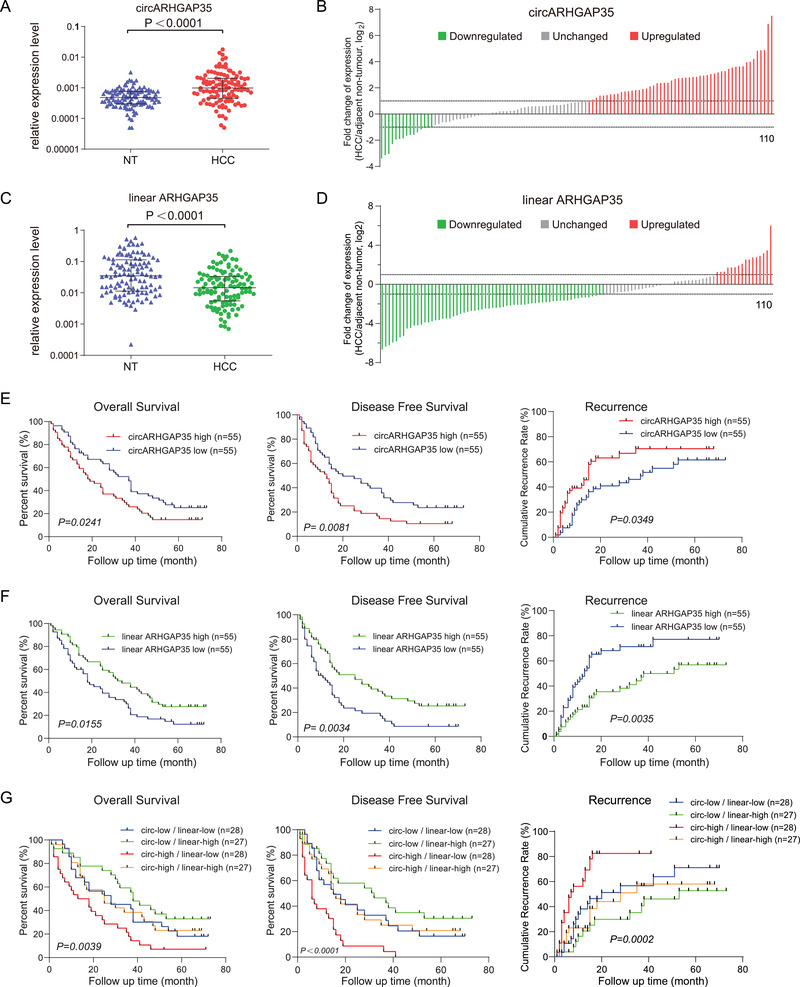
The upregulation of circARHGAP35 is associated with poor survival in cancer patients. A) The expression of circARHGAP35 in 110 paired HCC and adjacent non‐tumor (NT) liver tissues. Data were analyzed by paired Student's *t*‐test, *n* = 110. B) The fold change of circARHGAP35 expression in 110 paired HCC samples (downregulated, green; unchanged, gray; upregulated, red). C) The expression of linear ARHGAP35 in 110 paired HCC and adjacent non‐tumor (NT) liver tissues. Data were analyzed by paired Student's *t*‐test, *n* = 110. D) The fold change of linear ARHGAP35 expression in 110 paired HCC samples (downregulated, green; unchanged, gray; upregulated, red). E) Kaplan–Meier analysis of the correlation between circARHGAP35 expression and overall survival (OS), disease free survival (DFS), and recurrence in 110 HCC patients. F) Kaplan–Meier analysis of the correlation between linear ARHGAP35 RNA expression and OS, DFS, and recurrence in 110 HCC patients. G) The 110 HCC patients were divided into four groups according to the expression levels of circARHGAP35 and linear ARHGAP35 RNA. Kaplan–Meier analysis of the correlation between circARHGAP35/linear ARHGAP35 RNA expression and OS, DFS, and recurrence in 110 HCC patients. Log‐rank tests were used to determine the statistical significance for (E), (F), and (G).

To explore the potential clinical implications of circARHGAP35 in cancer, we detected the expression of circARHGAP35 and linear ARHGAP35 in a cohort of 110 paired HCC and adjacent non‐tumor tissues (Tables S1 and S2, Supporting Information). Notably, most of the HCC samples (85/110, 77.3%) were HBV related HCC. We observed that there was no significant difference in the expression of circARHGAP35 between HBV positive and HBV negative samples (Figure S7A–C, Supporting Information), indicating that circARHGAP35 might not be associated with HCC etiology. We found that circARHGAP35 was significantly upregulated and linear ARHGAP35 was downregulated in HCC (**Figure** **7**A,C). The upregulation of circARHGAP35 and downregulation of linear ARHGAP35 were observed in 47.3% (52/110) and 57.3% (63/110) of HCC samples, respectively (Figure 7B,D). Moreover, Kaplan–Meier survival analysis showed that the high abundance of circARHGAP35 in these samples was associated with shorter overall survival (OS), shorter disease free survival (DFS), and a higher recurrence rate (Figure 7E), while patients with high linear ARHGAP35 expression had a better outcome (Figure 7F). When we combined the expression of circARHGAP35 and linear ARHGAP35, patients with high levels of circARHGAP35 and low levels of linear ARHGAP35 had a significantly shorter OS, shorter DFS, and higher recurrence rate compared to those with low levels of circARHGAP35 and high levels of linear ARHGAP35 (Figure 7G). Similarly, we also found that circARHGAP35 was upregulated and linear ARHGAP35 was downregulated in a 62 paired colorectal cancer (CRC) patients' cohort (Figure S7D–G, Supporting Information). Previously, we had demonstrated that human blood extracellular vesicles (EVs) contained an abundance of circRNAs.^[^ ^30^ ^]^ We speculated that circARHGAP35 might be present in the blood EVs of cancer patients. Analysis in a cohort of 35 blood EV samples from HCC patients revealed a relatively high frequency of detection of circARHGAP35 expression (Figure S7H, Supporting Information).

## 3 Discussion

**Figure 8:**
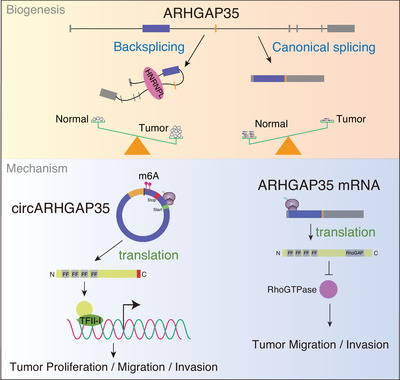
Integrated model depicting circARHGAP35 driving cancer progression. circARHGAP35 is derived from the locus of a tumor suppressor gene, ARHGAP35, and its biogenesis is regulated by HNRNPL. circARHGAP35 is translated into a large protein in an m^6^A‐dependent manner. The circARHGAP35 protein exerts its oncogenic roles by interacting with TFII‐I, while ARHGAP35 suppresses cancer cell motility by decreasing RhoA activity.

Recent studies indicate that circular RNAs are aberrantly expressed in human cancers or other cell types and that dysregulated circRNAs play an important role in diseases.^[^ ^13^, ^31^, ^32^ ^]^ However, the role of circRNAs in human cancers and related mechanisms remain largely unknown. In this study, we identified a novel circRNA termed as circARHGAP35, originating from the locus of the tumor suppressor gene ARHGAP35, and often upregulated in cancer tissues. Functional and mechanistic studies showed that HNRNPL facilitated circARHGAP35 biogenesis and that circARHGAP35 was translated into a large protein in an m^6^A‐dependent manner. We also found that the circARHGAP35 protein promoted tumor progression by interacting with TFII‐I, while ARHGAP35 inhibited cancer cell migration and invasion by limiting RhoA activity (**Figure** **8**). Our findings reveal a novel mechanism of oncogene activation by circRNA and provide valuable insights into the complexity of the cancer transcriptome.

Although numerous studies have shown that circRNAs function as microRNA sponges,^[^ ^13^, ^32^, ^33^ ^]^ it has been demonstrated that certain circRNAs get translated into proteins/peptides.^[^ ^16^, ^18^, ^34^, ^35^ ^]^ We evaluated the coding potential of circARHGAP35 and found that circARHGAP35 contained a 3867 nt ORF, spanning from an AUG start codon derived from a host gene to a UGA stop codon produced 44 nt beyond the splice junction. Compared with other peptides encoded by circRNAs, such as circPPP1R12A‐73aa in colon cancer^[^ ^34^ ^]^ and SHPRH‐146aa^[^ ^35^ ^]^ in glioblastoma, the protein encoded by circARHGAP35 is remarkably large (1289 aa). As circRNA‐derived peptides/proteins are often truncated versions of the cognate proteins, they are proposed to act as dominant‐negative protein variants, decoys, or modulators of alternate protein complexes.^[^ ^36^ ^]^ However, the substantially large molecular weight of circARHGAP35 confers multiple functional domains for its independent role, distinguishing it from other small peptides/proteins of circRNAs. Of note, the truncated translation phenotypically mimics genomic variations like nonsense mutations and frameshift indels which result in the early termination of translation. Although not confirmed in this study, we speculate that there might be protein‐coding circRNAs translated using the cognate internal start codons, which resemble an alternative transcription initiation that leads to the expression of a novel ALK isoform in cancer.^[^ ^37^ ^]^ Here, we prove that cancer cell lines can give rise to an oncogene without genetic and epigenetic aberrations. Consequently, we argue that the translation from a circRNA represents a novel mechanism for oncogene activation in cancer.

ARHGAP35, also known as p190‐A, is a member of the Rho GTPase activating protein (RhoGAPs) family, which “switches off” Rho GTPase by stimulating the GTP hydrolyzing activity of Rho and turning it to a GDP‐bound inactive state.^[^ ^38^ ^]^ Previous studies have shown that ARHGAP35 regulates cell cycle and metastasis, and acts as a tumor suppressor gene.^[^ ^22^, ^39^ ^]^ Consistent with previous reports, we have confirmed that ARHGAP35 reduces RhoA activity, decreases stress fiber formation, and suppresses cancer cell migration and invasion. ARHGAP35 protein contains four FF domains at the N‐terminus and a Rho GAP domain at the C‐terminus. The FF domain is mainly present in a variety of nuclear transcription and splicing factors,^[^ ^40^ ^]^ while ARHGAP35 and ARHGAP5 (p190‐B) are solely cytoplasmic proteins containing FF domains.^[^ ^41^ ^]^ The circARHGAP35 protein, which lacks the Rho GAP domain and has a distinct C‐terminus, exhibits nuclear localization like other FF domain‐containing proteins. We found that circARHGAP35 had no effect on RhoA activity and little effect on stress fiber formation, owing to the absence of the Rho GAP domain. A previous study suggested that the transcriptional regulator TFII‐I interacted with the FF domains of ARHGAP35, and this interaction diminished TFII‐I transcriptional activity, owing to the cytoplasmic sequestering of TFII‐I.^[^ ^24^ ^]^ However, in this study, the suppression of cancer cell motility by ARHGAP35 by binding to TFII‐I was unlikely because TFII‐I was almost exclusively found in the nucleus while ARHGAP35 was mainly localized in the cytoplasm. Nevertheless, we observed an interaction between TFII‐I and the circARHGAP35 protein. We have proven that TFII‐I was a key partner for circARHGAP35 protein to exert its oncogenic functions, thereby demonstrating the need for the circARHGAP35 protein by cancer cell lines to establish and maintain an oncogenic transcriptome (e.g., FOS). Elucidation of the complete set of targets and pathways regulated by the circARHGAP35/TFII‐I axis, and its effect on cancer parthenogenesis requires further investigation. Our findings on the distinct roles of circARHGAP35 and ARHGAP35 represent a new type of intragenic regulon (iRegulon), initially reported by Guarnerio et al., which performs diverse and antithetical biological functions through linear and circular RNA products.^[^ ^11^ ^]^

Notably, our results showed that circARHGAP35 was upregulated in cancer, while its cognate ARHGAP35 mRNA was downregulated. Further, we speculated that certain trans‐acting factors participated in the biogenesis of circARHGAP35. Increasing evidence has demonstrated that RBPs including QKI,^[^ ^8^ ^]^ HNRNPL,^[^ ^27^ ^]^ MBL,^[^ ^14^ ^]^ and NF90/NF110,^[^ ^42^ ^]^ regulate the circularization of exons by binding to specific motifs on the flanking introns and promoting backsplicing of circRNAs. Moreover, Xiang Li et al., reported 103 RBPs which affected circularization.^[^ ^42^ ^]^ Our siRNA screening, which targeted 63 RBPs deregulated in HCC^[^ ^33^ ^]^ indicated that HNRNPL was involved in circARHGAP35 biogenesis. HNRNPL, which is often up‐regulated in many types of solid cancer (TCGA data), plays an important role in the biogenesis of circARHGAP35, suggesting that transcriptome alterations mediated by abnormal RBPs during cancer activation and progression may give rise to oncogene activation in a way that does not rely on genomic or epigenomic variations. Future study is warranted to elucidate the targets and pathways regulated by the HNRNPL‐circARHGAP35‐TFII‐I axis in cancers. We acknowledge that we have not completely addressed the deregulation of circARHGAP35 and its cognate mRNA in cancer. It is possible that ARHGAP35 mRNA might be regulated by miRNAs targeting the 3′UTR of the mRNA.^[^ ^43^ ^]^ Further research is needed to completely elucidate transcriptional regulation, processing, and turnover of the transcriptional output of this locus.

## 4 Conclusion

In summary, we have characterized a novel circular RNA derived from the tumor suppressor gene, ARHGAP35. Our data demonstrates that circARHGAP35 is translated into a large oncogenic protein in cancer cell lines, while its cognate linear mRNA encodes a tumor suppressor. Importantly, our study expands current knowledge on oncogene activation by circRNA in cancer pathogenesis and the complexity of the cancer transcriptome.

## 5 Experimental Section

##### RNA‐seq Analysis

The RNA‐seq of 12 paired HCC and adjacent cancer tissues 12 HCC tissues were performed by ribosomal RNA‐depleted total RNA sequencing. The total RNA samples (3 µg) were treated with the RiboMinus Eukaryote Kit (Qiagen, Valencia, CA) to remove ribosomal RNA. Strand‐specific RNA‐seq libraries were prepared using the NEBNext Ultra Directional RNA Library Prep Kit for Illumina (NEB, Beverly, MA) according to the manufacturer's instructions. The raw sequencing reads were filtered by FastQC, and aligned using the spliced read aligner TopHat2. circRNAs from unmapped reads were discovered by a circRNA identification software package circ_find. The relative expression of a circRNA was denoted as spliced reads per billion mapped reads. The BEDtools coverage tool was used to annotate circRNA position and calculate exonic circRNA length by intersecting with GENCODE V29 reference. T‐distributed stochastic neighbor embedding (t‐SNE) was conducted for multidimensional scaling analysis using the R package Rtsne.

##### Samples Collection

Human primary HCC, colorectal cancer, and adjacent non‐tumorous tissues were collected from the biopsy or surgery. The participants in the study provided informed consent, and the study was approved by the Clinical Research Ethics Committee of Fudan University Shanghai Cancer Center, China. 50 paired HCC and the corresponding adjacent non‐tumor liver tissues were obtained from the TCGA database (https://cancergenome.nih.gov/).

##### RNA Preparation and Quantitative Reverse Transcription Polymerase Chain Reaction (qRT‐PCR)

The total RNA was extracted using the TRIzol reagent (Life Technologies, Carlsbad, CA, USA). Complementary DNA was synthesized using the PrimeScript RT reagent kit (TaKaRa, Tokyo, Japan) using 500 ng total RNA as template in a final volume of 10 µl. The qRT‐PCR was carried out using 0.6 µl of the reverse‐transcription reaction solution with SYBR Premix Ex Taq (TaKaRa) in a final volume of 20 µl in 7900 Real‐Time PCR System (Applied Biosystems). *β*‐actin was used as an internal control. The primers used are listed in Table S3, Supporting Information.

##### Cell Culture and Treatments

HEK‐293T, SK‐Hep‐1, and HCT‐116 cells were obtained from American Type Culture Collection (ATCC). HuH‐7 cells were obtained from the Japanese Collection of Research Bioresources (Tokyo, Japan). HEK‐293T, SK‐Hep‐1, and HuH‐7 cells were cultured in a humidified incubator at 37 °C with 5% CO_2_ in Dulbecco's modified Eagle's medium (DMEM) supplemented with 10% fetal bovine serum (FBS) (HyClone, Logan, UT, USA), 100 U mL^−1^ penicillin and 100 µg mL^−1^ streptomycin (Invitrogen, CA, USA). HCT‐116 cells were cultured in McCoy's 5A Modified medium containing 10% FBS and 1 × penicillin‐streptomycin at 37 °C with 5% CO_2_. All cells used in our study were tested for mycoplasma contamination and were authenticated by short tandem repeats (STR) sequencing. Transcription was blocked by adding 1 µg mL^−1^ actinomycin D or dimethyl sulfoxide (Sigma‐Aldrich, St Louis, MO) to the cell culture medium. Total RNA (2 µg) was incubated for 20 min at 37 °C with or without 3 U µg^−1^ RNase R (Epicentre Technologies, Madison, WI, USA).

##### Fluorescence in Situ Hybridization (FISH)

The Cy3‐labelled probes spanning the splice junction specific to circARHGAP35 were designed and synthesized by RiboBio (Guangzhou, China). The assay was performed using RiboTM Fluorescent In Situ Hybridization Kit (Ribobio Company, China). Briefly, cells were seeded onto glass slides (Merck Millipore) and then fixed with 4% paraformaldehyde for 10 min at room temperature. Cells were then permeabilized with 0.5% Triton X‐100 for 5 min at 4 °C. After three washes for 5 min, cells were blocked with a pre‐hybridization buffer for 30 min at 37 °C. Then the cells were incubated in hybridization buffer with a FISH probe at 37 °C in the dark overnight. After three times with Wash Buffer I (4 × SSC with 0.1% Tween‐20), once with Wash Buffer II (2 × SSC), Wash Buffer III (1 × SSC) at 42 °C in the dark for 5 min and one wash with 1 × PBS at room temperature, the cells was mounted with Prolong Gold Antifade Mountant with DAPI (Thermo Fisher Scientific). Images were acquired using an Olympus FluoView FV1000 confocal microscope.

##### Northern Blotting

The authors performed northern blotting using NorthernMax Kit (Thermo Fisher Scientific, Carlsbad, California, USA). Briefly, RNA (15 µg for detection of endogenous circARHGAP35 and 8 µg for detection of linear ARHGAP35) was denatured with 3 volumes formaldehyde load dye (Ambion) for 15 min at 65 °C and loaded on 1% agarose gel. After the electrophoresis, RNA was transferred on Hybond N+ membrane (GE Healthcare, Uppsala, Sweden) by capillary transfer. Transferred RNA was ultraviolet‐crosslinked (at 265 nm) at 200000 µJ cm^−2^. Pre‐hybridization was performed at 68 °C for 30 min and hybridization was performed at 68 °C overnight. The membrane was washed with 2× SSC 0.1% SDS twice 5 min at room temperature, then twice 15 min with 0.1× SSC 0.1% SDS at 68°C. The membrane was hybridization with anti‐DIG antibody and washed using DIG Wash and Block Buffer Set (Roche, Indianapolis, IN, USA). After washing, the blot was detected with the DIG luminescence detection kit (Roche). DIG‐labeled probes were prepared using DIG Northern starter Kit (Roche) by in vitro transcription with PCR products as templates for T7 transcription.

##### Subcellular Fractionation

Subcellular fractionation, to obtain cytoplasmic and nuclear fractions, was performed using the NE‐PER Nuclear and Cytoplasmic Extraction Reagents (Thermo Fisher Scientific, Carlsbad, California, USA) according to the manufacturer's instructions. *β*‐actin was used as the cytoplasmic endogenous control. U2 small nuclear RNA was used as the nuclear endogenous control.

##### Oligonucleotide Transfection

The small interfering RNA (siRNA) oligonucleotides and the negative control siRNA were synthesized by Ribobio (RiboBio Biotechnology, Guangzhou, China). Cells were transfected with Lipofectamine RNAiMax (Life Technologies, Carlsbad, CA, USA) and incubated for 48 h. Following the incubation period, they were collected for other assays. The sequences used are shown in Table S5, Supporting Information.

##### Lentivirus Production and Infection

HEK‐293T cells were transfected with p‐circARHGAP35, p‐circARHGAP35‐Flag, p‐lin‐cORF‐Flag, p‐lin‐cORF‐Flag‐mut, lentiGuide‐puro‐gRNA, along with the packaging and envelope plasmids psPAX2 and pMD2.G, respectively (gifts from Dr. Didier Trono) using Lipofectamine 2000 (Life Technologies, Carlsbad, CA, USA) according to the manufacturer's instructions. Virus particles were harvested 48 h after transfection. HuH‐7, SK‐Hep‐1, and HCT‐116 cells were infected with lentivirus plus 6 µg mL^−1^ polybrene (Sigma‐Aldrich, St. Louis, MO, USA).

##### CRISPR/Cas9 Experiments

Linear ARHGAP35 transcript was specifically knocked down by the CRISPR/Cas9 technology. The Cas9 cutting site was in Exon5 of ARHGAP35 (not in the region that forms circARHGAP35). The sequence of gRNA was searched in http://crispr.mit.edu/ and presented in Table S3, Supporting Information. The gRNA sequence of ARHGAP35 was cloned into lentiGuide‐Puro (Addgene #52963) to generate lentiGuide‐Puro‐gRNA35. Then the lentivirus of lentiGuide‐Puro, lentiGuide‐Puro‐gRNA35, and lentiCas9‐Blast (Addgene #52962) was collected. HuH‐7 cells were infected with lentiCas9‐Blast lentivirus for 48 h and treated with 4 µg ml^−1^ blasticidin (Life Technologies), and the lentiGuide‐Puro‐gRNA35 or lentiGuide‐Puro lentivirus was added to the cells for 48 h and treated with 4 µg ml^−1^ puromycin (Sangon Biotech, Shanghai, China). The expression of ARHGAP35 protein was verified by Western blotting.

##### Cell Proliferation and Colony Formation Assays

Cell proliferation assay was performed with Cell Counting Kit‐8 (Dojindo Laboratories, Kumamoto, Japan). Cells were seeded at a density of 1000 cells per well in 96‐well plates. Ten microliters of CCK‐8 solution were added into each well. After 2 h of incubation at 37 °C, the absorbance at 450 nm was measured. Each measurement was performed in triplicate and the experiments were repeated at least three times. For the colony formation assays, cells were trypsinized and seeded in 6‐well plates at a density of 1000 cells per well. After 14 days, colonies were dyed with solution containing 0.1% crystal violet and 20% methanol, and then imaged using an IX71 inverted microscope (Olympus, Tokyo, Japan).

##### Cell Migration and Invasion Assays

For the migration assays, 5 × 10^4^ cells were seeded into the upper chamber of each 8‐µm pore size transwell insert (BD Biosciences, Franklin Lakes, NJ, USA). For the invasion assay, 1 × 10^5^ cells were placed in the upper chamber of each Matrigel‐coated insert. DMEM supplemented with 10% FBS was used as the chemoattractant in the lower chamber. After incubation at 37 °C, cells remaining in the upper chamber were removed with cotton swabs, and cells adhering to the lower membrane were stained with 0.1% crystal violet in 20% methanol. The cells that had migrated or invaded to the basal side of the membrane were imaged and counted using an IX71 inverted microscope (Olympus, Tokyo, Japan).

##### Animal Experiments

For the in vivo tumorigenicity assay, 2 × 10^6^ cells were injected subcutaneously into the flank region of each nude mouse (males, 4 weeks old). After transplantation, the mice were monitored weekly for tumor size. After 8 weeks, the mice were sacrificed. The tumors were fixed and prepared for histological examination. For in vivo metastasis assay, 2 × 10^6^ cells were injected into the tail veins of nude mice. About 2 months later, the mice were sacrificed and the lung tissues were fixed, paraffin‐embedded, and sectioned for histopathological examination. All experiments were performed in accordance with protocols approved by Fudan University Experimental Animal Care Commission.

##### Western Blot Analysis

The proteins were separated by sodium dodecyl sulfate polyacrylamide gel electrophoresis (SDS‐PAGE) and transferred to a nitrocellulose membrane (Bio‐Rad, Hercules, CA, USA). The membrane was blocked using 5% non‐fat milk and incubated with primary antibodies. The immune complexes formed were detected using enhanced chemiluminescence reagents (Pierce, Rockford, IL, USA). The antibody against ARHGAP35 was obtained from Cell Signaling Technology, and the antibody against FLAG was obtained from Sigma. Information on the antibodies is listed in Table S4, Supporting Information.

##### Vector Construction

For the circRNA expression vector (p‐circARHGAP35), the genomic region for circARHGAP35 was amplified from HEK293T genomic DNA using PrimerSTAR Max DNA Polymerase Mix (Takara) and cloned into circular RNA expression plasmid PLCDH‐ciR (Geenseed Co, Ltd, Guangzhou, China). p‐circARHGAP35‐Flag was derived by inserting 3 × Flag sequence immediately to the upstream of stop codon of circARHGAP35. The circARHGAP35 ORF sequence or mutatant sequence (the stop codon TGA was deleted) was amplified and cloned into pCDH‐CMV‐MCS‐EF1‐Puro (SBI, Palo Alto, CA, USA) to generate p‐lin‐cORF‐Flag and p‐lin‐cORF‐Flag‐mut. p‐lin‐FL was produced by inserting a full length of ARHGAP35 ORF sequence into pCDH‐CMV‐MCS‐EF1‐Puro vector. The gRNA sequence targeting ARHGAP35 were designed and cloned into lentiGuide‐puro vector (a generous gift from Dr. Feng Zhang). pcDNA5‐FTO‐HA was a generous gift from Dr. Zefeng Wang. pTRIPZ‐circARHGAP35‐ORF was constructed by inserting the Flag‐tagged circARHGAP35 ORF sequence into the pTRIPZ vector (Open Biosystems, Huntsville, USA), a Tet‐On lentiviral expression vector. The two HNRNPL binding sites sequences were synthesized (GENEWIZ, Suzhou, China), and together with the GFP sequence were cloned into PCDH‐CMV‐MCS‐EF1‐Puro vector. The primers are all listed in Table S3, Supporting Information. All constructs were verified by sequencing.

##### GST Pull Down

HEK‐293T cells were cultured in 100‐mm culture dish in DMEM supplemented with 10% FBS. Then 6 µg p‐MS2‐circARHGAP35 plasmid or 6 µg control plasmid p‐MS2 and 4 µg pCDH‐MS2‐GST‐Puro‐NES (containing MS2 binding protein which recognized MS2 RNA, GST tag which recognized glutathione‐SH, and a cytoplasmic localization signal) were transfected in HEK‐293T cells using lipofectamine 2000 (Life Technologies). Forty‐eight hours later, cells were washed twice with PBS and lyse in 800 µl lysis buffer containing 20 mm Tris‐HCl at pH 7.5, 100 mm KCl, 5 mm MgCl_2_, 0.5% NP‐40, protease inhibitors (Thermo Fisher Scientific), RNase inhibitor (TaKaRa), and 10 mm DTT for 10 min on ice. The cell lysates were collected by scraping and centrifuged at 10000g for 15 min at 4 °C. The supernatant was collected. Then supernatant was incubated with 30 µl MagneGST Particles (Promega, Madison, WI) for 30 min at room temperature on a rotating platform. After five washes, 1 × SDS loading buffer was added. The protein complexes obtained were separated by SDS‐PAGE and analyzed by mass spectrum.

##### Polysome Profiling

HCT‐116 cells grow in 100 mm dishes to ≈80% confluence. Cells were treated with 100 µg mL^−1^ cycloheximide and incubated at 37 °C for 3–4 min. Cells were then lysed in 300 mL of lysis buffer containing 10 mM NaCl, 10 mM MgCl_2_, 10 mM Tris‐HCl pH 7.5, 1% Triton X‐100, 1% sodium deoxycholate, 0.2 U µL^−1^ RNase inhibitor, 1 mM DTT, and 0.1 mg mL^−1^ cycloheximide. Immediately the samples were placeed on ice for 2 min and then centrifuged for 5 min at 16 000 × g at 4 °C to pellet the nuclei and cellular debris. For EDTA treatments, 50 mm EDTA final was added to cell lysate and incubated for 10 min on ice just before applying on gradient before ultracentrifugation. Linear sucrose gradients were prepared with a Gradient Master (Biocomp). Cytoplasmic lysates were loaded into 15% to 50% sucrose gradients and separated by ultracentrifugation with a SW41 rotor (Beckman) at 274000 × g for 1 h 40 min at 4 °C. Fractions were collected with a BioComp Piston Gradient Fractionator equipped with a Bio‐Rad Econo UV Monitor (set at 254 nm). RNA extracted from each fraction was extracted with Trizol‐LS (Thermo Fisher Scientific) following the manufacturer's instruction and analyzed by qRT‐PCR. The sum of all fractions was considered as total of one RNA.

##### m^6^A Immunoprecipitation

Total RNA was extracted from cells using the TRIzol reagent. The RNA obtained was treated with DNase I for 20 min at 37 °C, to remove DNA contamination. About 5 µg of total RNA was fragmented for 5–6 min at 70°C. About 30 µl of protein‐G magnetic beads (Thermo Fisher Scientific) was washed twice using IP buffer (150 mm NaCl, 10 mm Tris‐HCl, pH 7.5, 0.1% IGEPAL CA‐630) and incubated with 3 µg of anti‐m^6^A antibody (Millipore, Germany) or IgG antibody (Sigma) in 200 µl of IP buffer at 4 °C for at least 6 h. Following two washes with IP buffer, the antibody‐bead complex was resuspended in 200 µl of the IP reaction mixture containing fragmented total RNA and RNasin Plus RNase Inhibitor (Promega, Madison, WI), and incubated for 2 h at 4 °C. The RNA reaction mixture was washed twice in IP buffer, twice in low‐salt IP buffer (50 mm NaCl, 10 mm Tris‐HCl, pH 7.5, 0.1% IGEPAL CA‐630), and twice in high‐salt IP buffer (500 mm NaCl, 10 mM Tris‐HCl, pH 7.5, 0.1% IGEPAL CA‐630) for 10 min each, at 4 °C. The bound RNAs were isolated using the RNeasy Mini Kit (Qiagen, Valencia, CA) and analyzed by qRT‐PCR.

##### Immunoprecipitation Assay

The cells were trypsinized, washed in PBS, lysed using an equal volume (as the cell pellet) of the lysis buffer (250 mm NaCl, 50 mm Tris pH 8, 5 mm EDTA, 0.8% NP40, 1 mm DTT, 5% glycerol, 50 mm NaF) containing protease inhibitors (Life Technologies), and centrifuged at 16400 × g for 15 min. The supernatants were incubated with anti‐FLAG antibody (Sigma‐Aldrich Aldrich) at 4 °C overnight, with gentle rotation. Subsequently, the antibody‐protein complexes were incubated with 30 µl of protein G Dynabeads (Life Technologies) for 2 h at 4 °C. The protein complexes obtained were separated by SDS‐PAGE and analyzed by MS and Western blot analysis.

##### RhoA Activity Assays

SK‐Hep‐1 cells were infected with pCDH‐ORF and pCDH‐FL lentivirus and empty control virus. At 48 h after transfection, the cells were harvested for the Rhotekin pulldown assays to test the active Rho. A Rho Activation Assay Biochem Kit (Cytoskeleton, Inc.) was used according to the manufacturer's protocol. Briefly, cells were placed on ice and rinsed with ice cold PBS to remove serum proteins. Cells were then lysed with Lysis Buffer containing 50 mm Tris pH 7.5, 10 mm MgCl_2_, 0.5 m NaCl, 2% IGEPAL, and protease inhibitor cocktail. Lysates were clarified by centrifugation at 10000 × g at 4 °C for 1 min, transferred to new tubes and measured protein concentrations. About 400 µg lysate was added to 50 µg rhotekin‐RBD beads and incubated at 4 °C on a rotator for 1 h. The beads were washed once with 500 µl Wash Buffer containing 25 mm Tris pH 7.5, 30 mm MgCl_2_, and 40 mm NaCl. 20 µl of 2 × Laemmli sample buffer was added to each sample. The activation of RhoA was analyzed by SDS‐PAGE and Western blotting with a 1:500 dilution of anti‐RhoA antibody.

##### Fluorescence Labeling and Imaging

Cells were seeded on glass culture slides (BD Falcon, Franklin Lakes, New Jersey, USA) and cultured for 24 h. Subsequently, they were fixed with 4% paraformaldehyde, permeabilized with 0.25% Triton X‐100, and blocked with 1% BSA. For cytoskeleton detection, F‐actin was detected with phalloidin‐Alexa Fluor 488 (Thermo Fisher Scientific, Carlsbad, California, USA). For the detection of protein localization, immunostaining was performed with FLAG (1:100), ARHGAP35 (1:100), and TFII‐I (1:100) primary antibodies, and subsequently the appropriate secondary antibodies. The nuclei were stained with 4′,6‐diamidino‐2‐phenylindole (DAPI) (Thermo Fisher Scientific). Images were acquired using an Olympus FluoView FV1000 confocal microscope.

##### Statistical Analysis

All data were represented as mean ± standard error of the mean (SEM) of at least three biological replicates. *p* Values were determined by using two‐tailed Student's *t*‐test or ANOVA test as indicated in corresponding figure legends. Statistical analyses were performed using GraphPad Prism 8 (GraphPad Software, Inc., San Diego, CA, USA) and SPSS v.20.0 (SPSS Inc., Chicago, IL, USA). Differences with *p* < 0.05 were considered as statistically significant and were noted by asterisks (*, *p* < 0.05; **, *p* < 0.01; ***, *p* < 0.001).

## Conflict of Interest

The authors declare no conflict of interest.

## Supporting information



Supporting Information

## Data Availability

Research data are not shared.
