# Supplementary material for: HNRNPL Circularizes ARHGAP35 to Produce an Oncogenic Protein
Source: Adv Sci (Weinh). 2021 May 1;8(13):2001701. doi: 10.1002/advs.202001701 (PMC8261482; doi:10.1002/advs.202001701)
Supplement: Supplementary file 1 — Supporting Information [file ADVS-8-2001701-s001.pdf]

WILEY-VCH

© 2021 Wiley-VCH GmbH

ADVANCED SCIENCE
Open Access

Supporting Information

for *Adv. Sci.,* DOI: 10.1002/advs.202001701

HNRNPL Circularizes ARHGAP35 to Produce An Oncogenic Protein

Yan Li, Bing Chen, Jingjing Zhao, Qin Li, Siyuan Chen, Tianan Guo, Yuchen Li, Hongyan Lai, Zhiao Chen, Zhiqiang Meng, Weijie Guo, Xianghuo He,* and Shenglin Huang**

Supporting Information

HNRNPL Circularizes ARHGAP35 to Produce An Oncogenic Protein

Yan Li, Bing Chen, Jingjing Zhao, Qin Li, Siyuan Chen, Tianan Guo, Yuchen Li, Hongyan Lai, Zhiao Chen, Zhiqiang Meng , Weijie Guo, Xianghuo He,* and Shenglin Huang**

Yan Li, Bing Chen, Jingjing Zhao, Qin Li, Siyuan Chen, Tianan Guo, Yuchen Li, Hongyan Lai, Zhiao Chen, Zhiqiang Meng, Weijie Guo, Xianghuo He,* and Shenglin Huang**

Dr. Y. Li, B. Chen, J. Zhao, Q. Li, Y. Li, H. Lai, Dr. W. Guo, Prof. S. Huang
Department of Integrative Oncology
Fudan University Shanghai Cancer Center
the Shanghai Key Laboratory of Medical Epigenetics
Institutes of Biomedical Sciences
Shanghai Medical College
Fudan University
Shanghai 200032, China
E-mail: slhuang@fudan.edu.cn; guoweijie1986@gmail.com

Dr. Z. Chen, Prof. X. He
Fudan University Shanghai Cancer Center and Institutes of Biomedical Sciences
Shanghai Medical College
Fudan University
Shanghai 200032, China
E-mail: xhhe@fudan.edu.cn

S. Chen
Department of Medical Oncology
Fudan University Shanghai Cancer Center
Shanghai Medical College
Fudan University
Shanghai 200032, China

T. Guo,
Department of Colorectal Surgery
Fudan University Shanghai Cancer Center
Shanghai Medical College
Fudan University
Shanghai 200032, China

Supplementary Figures and Figure Legends

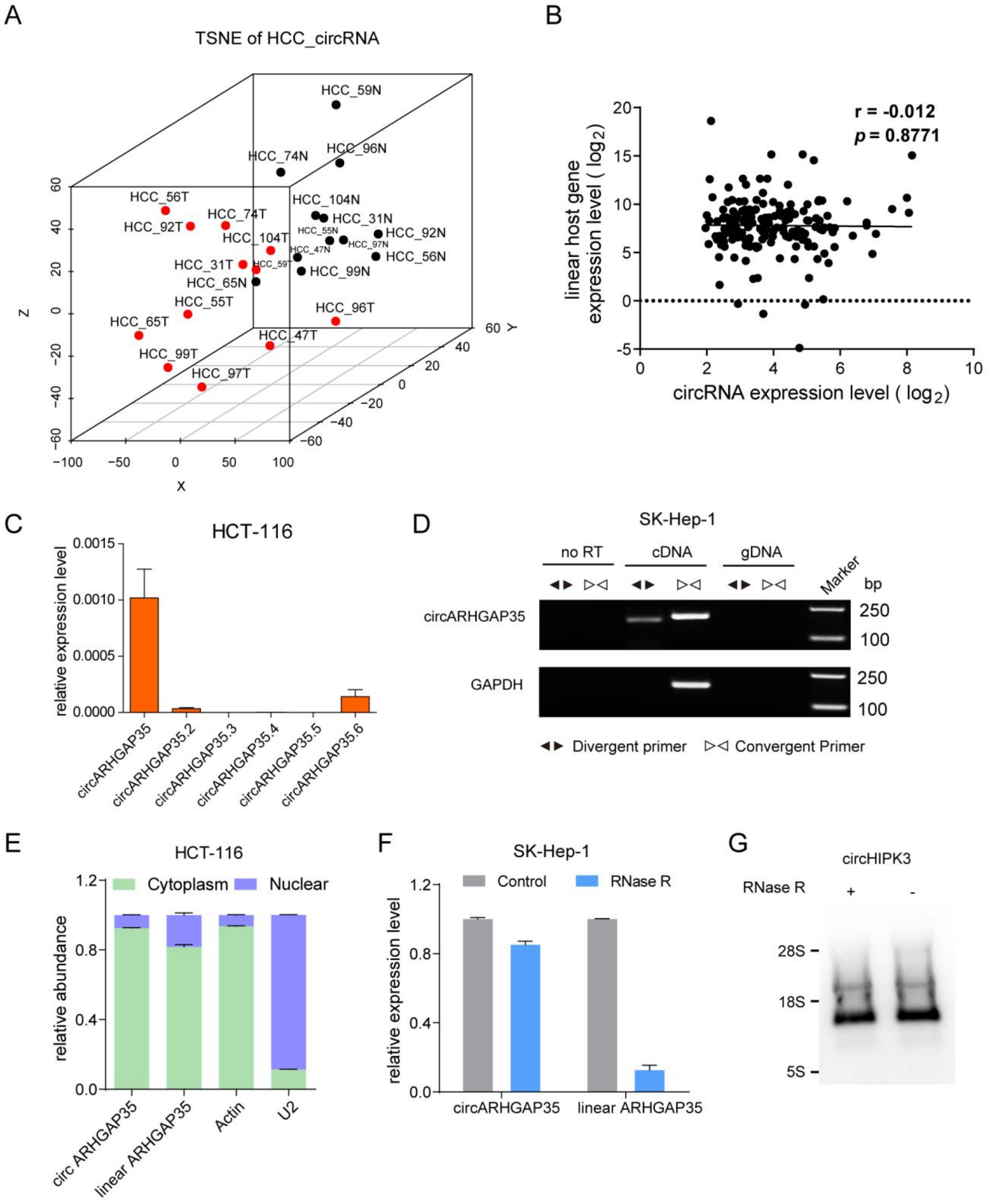

Figure S1. Identification of circRNAs in cancer tissues. A) Multidimensional scaling analysis for circRNAs in tumor (T) tissues and adjacent non-tumor (N) tissues. Red dots and black dots represented circRNAs in tumor tissues and adjacent non-tumor tissues, respectively. B) The Pearson's correlation of circRNA versus linear host gene expression. C) The expression of six circular ARHGAP35 isoforms in HCT-116 cells. Data were represented as mean ± SEM. D) Validation for the formation of circARHGAP35 in SK-Hep-1 cells . Divergent primers amplified the circular RNA in cDNA but not in genomic DNA (gDNA). Convergent primers spanning exons were used to detecte specific linear RNA. GAPDH was used as a

linear RNA control. E) qRT-PCR analysis of circARHGAP35 and linear ARHGAP35 RNA expression in the cytoplasm or nucleus of HCT-116 cells. F) qRT-PCR analysis of circARHGAP35 and linear ARHGAP35 RNA expression in the cytoplasm or nucleus of SK-Hep-1 cells. G) Northern blot for circHIPK3, which we reported previously (Zheng, et al., Nat Commun. 2016, PMID: 27050392), as a positive control, with or without RNase R treatment using a specific probe.

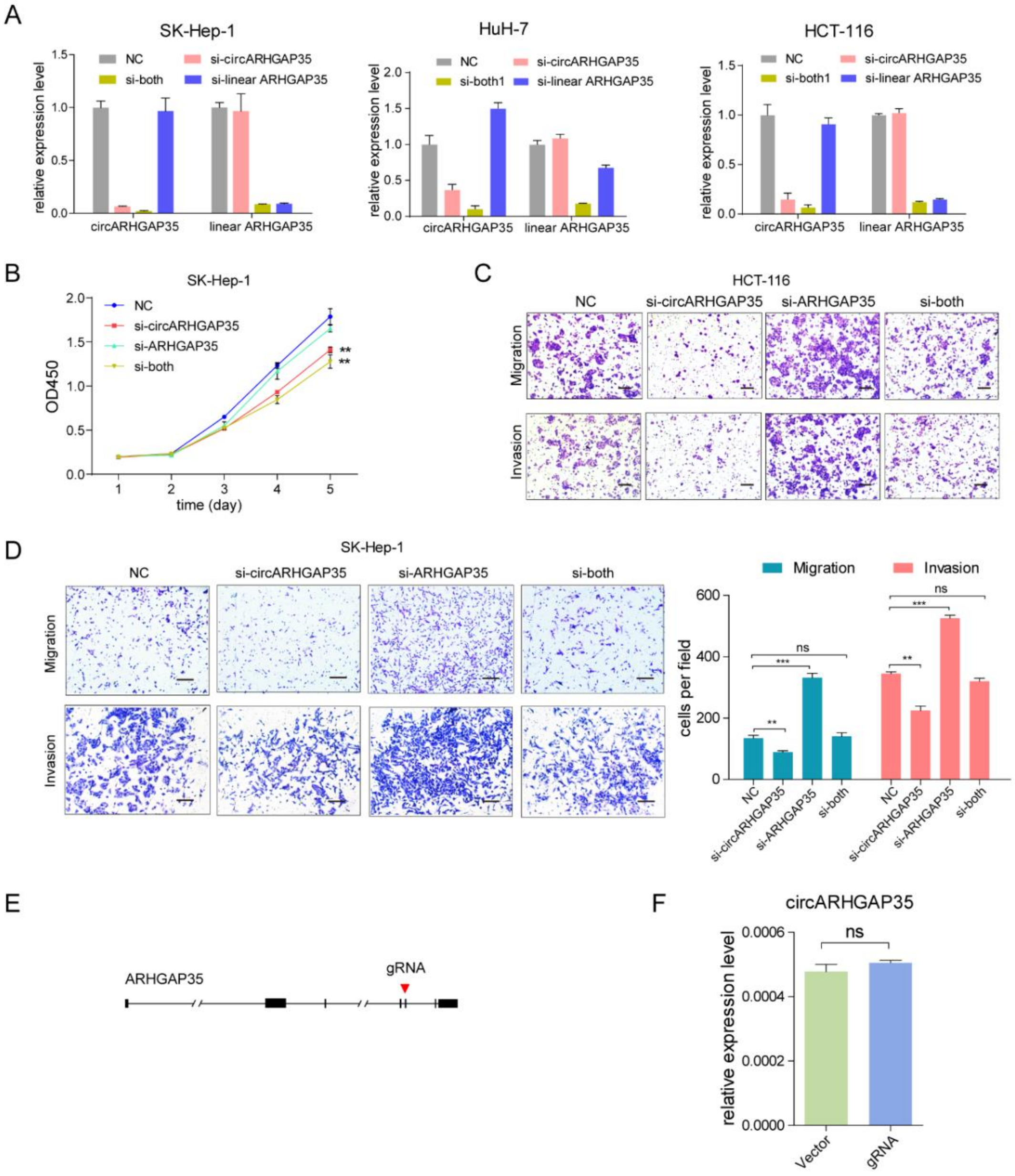

Figure S2. circARHGAP35 and linear ARHGAP35 have antithetical functions *in vitro*.**(A)** qRT-PCR analysis of the interference efficiencies after treatment with the siRNAs specifically targeting the back-splice junction of circARHGAP35, the ARHGAP35 linear transcript and both transcript in SK-Hep-1, HuH-7 and HCT-116 cells. **(B)** CCK-8 proliferation assay of SK-Hep-1 cells transfected with control or indicated siRNAs. **(C, D)** Transwell migration and invasion assays of HCT-116 (C) and SK-Hep-1(D) cells were performed after transfection of control or indicated siRNAs. Scale bars, 10 μm. **(E)** The position of gRNA targeting linear ARHGAP35 transcript. **(F)** The expression of circARHGAP35 after linear ARHGAP35 transcript was specifically knocked down by the CRISPR/Cas9 technology. Data were represented as mean ± SEM and representative of at least 3 independent experiments. Two-way ANOVA and Tukey post hoc test were performed for (B); one-way ANOVA and Dunnett post hoc test were performed for (D); unpaired Student's t tests were performed for (F). $^{*}P < 0.05$; $^{**}P < 0.01$; $^{***}P < 0.001$.

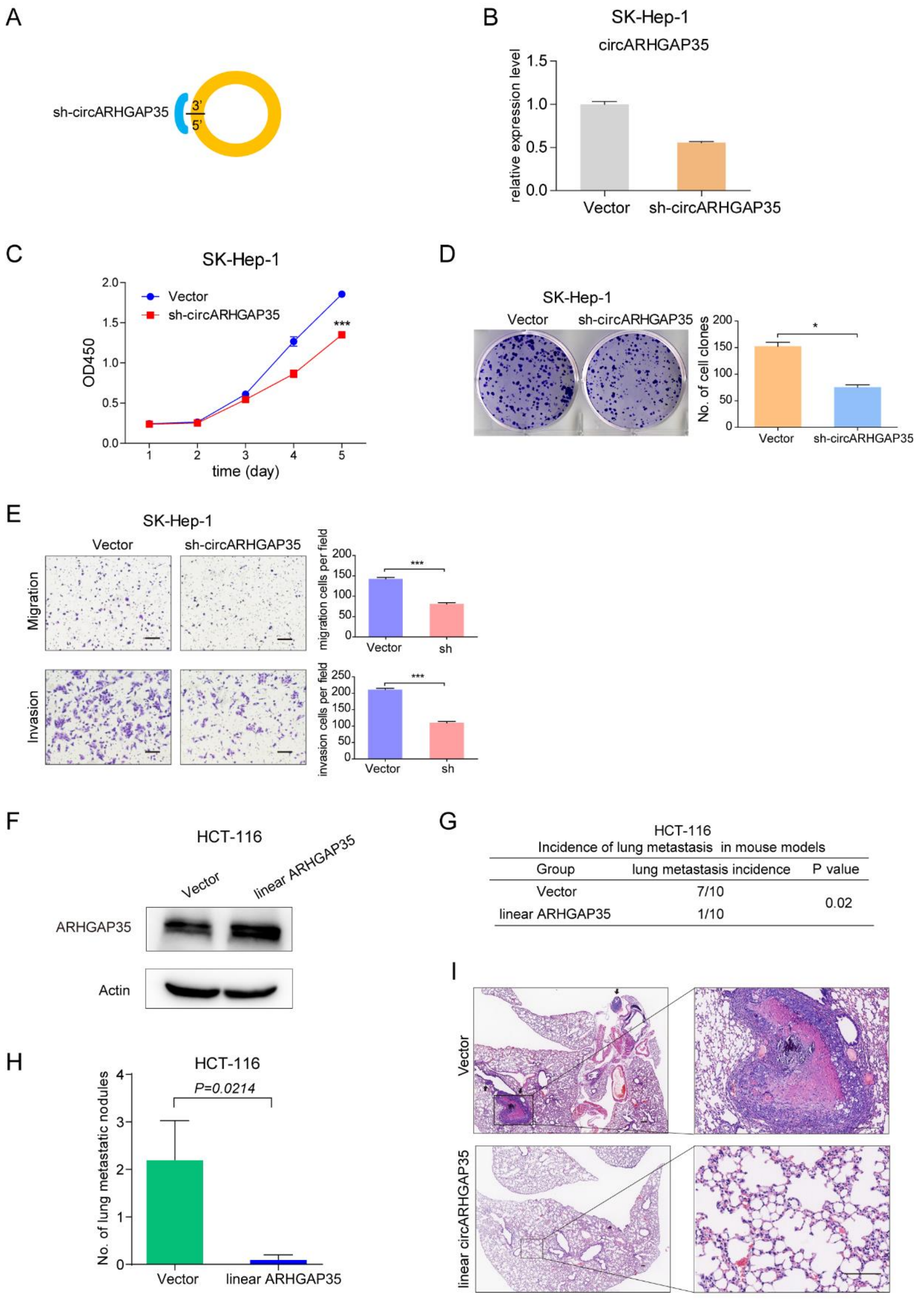

Figure S3. The functions of circARHGAP35 and linear ARHGAP35 *in vitro* and *in vivo.* A) Shematic representation of shRNA targeting circARHAGP35 at the back-splice junction. B) The relative expression of circARHGAP35 was determined by qRT-PCR in SK-Hep-1 cells infected with either shRNA expressing lentivirus or vector control lentivirus. C) CCK-8

proliferation assay in SK-Hep-1 cells after knockdown of circARHAGP35 by shRNA. D) Cloning formation assay after knockdown of circARHGAP35 by shRNA. E) Transwell migration and invasion assays of SK-Hep-1 cells after knockdown of circARHGAP35 by shRNA. Scale bars, 10 μm. F) Western blot analysis of ARHGAP35 protein level in HCT-116 cells infected with either linear ARHGAP35 expressing lentivirus or vector control lentivirus. G) The effect of linear ARHGAP35 on tumor metastasis in mouse tail vein injection model. The linear ARHGAP35 overexpression group or vector control group were injected into the tail vein of each nude mouse (n=10). Statistical analysis of the differences between the two groups was performed using the $\chi 2$ test. H,I) Hematoxylin-eosin-stained sections of lung metastatic nodules formed in the two groups. Slides were examined by an expert pathologist. The number of metastatic nodules in the lungs of the two groups were counted and analyzed (H). Black arrows indicate the nodules formed in the lung (I). Scale bars, 200 μm. Shown were representative images. Data were represented as mean ± SEM and representative of at least three independent experiments. Two-way ANOVA and Tukey post hoc test were performed for (C); one-way ANOVA and Dunnett post hoc test were performed for (D, E); unpaired Student's t tests were performed for (H). *$P < 0.05$; **$P < 0.01$; ***$P < 0.001$.

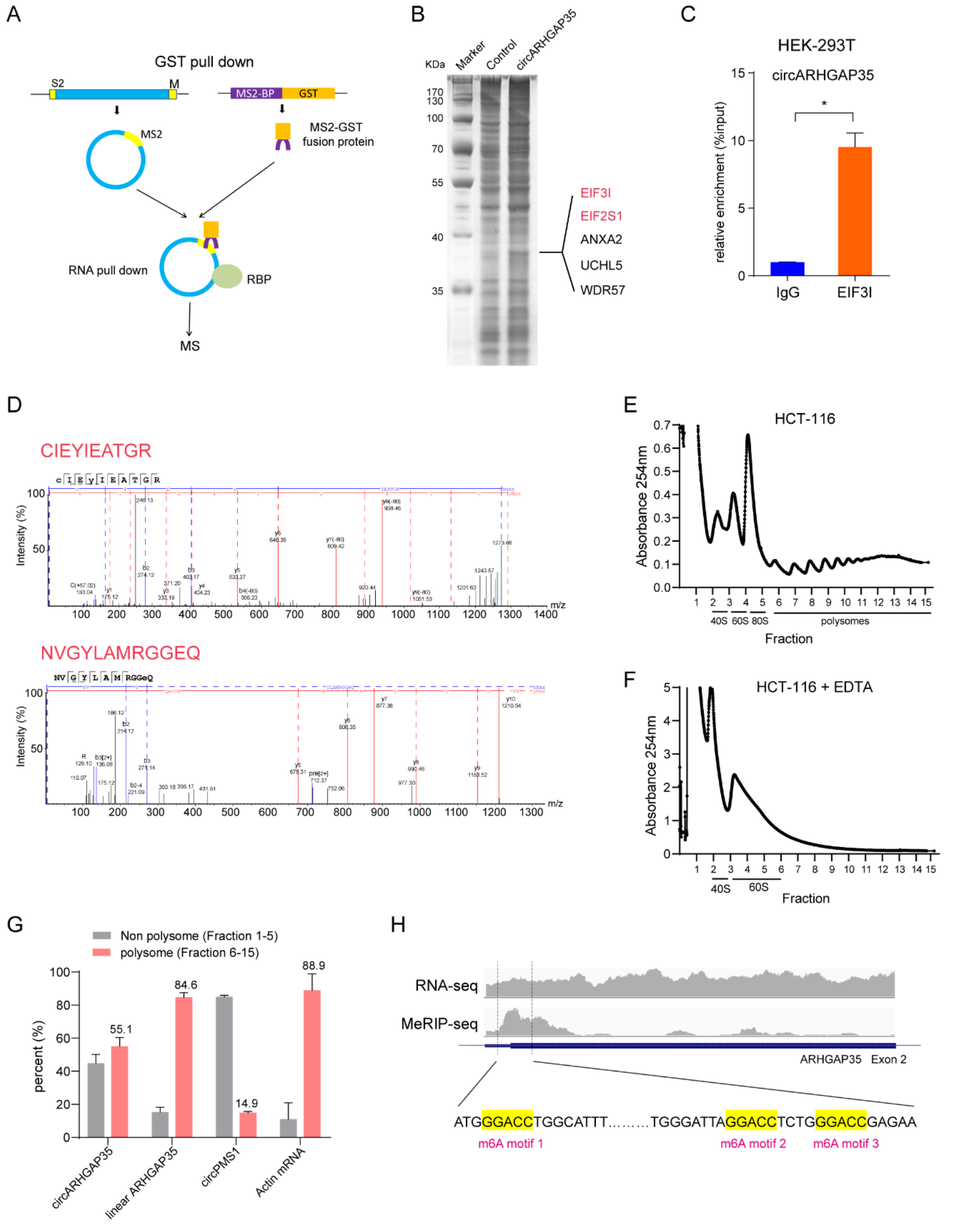

Figure S4. circARHGAP35 encodes a protein. A) Schematic of circARHGAP35 MS2-GST RNA pulldown assay. circARHGAP35 was tagged with MS2 hairpins.The plasmid p-MS2-circARHGAP35 or control plasmid was co-transfected with plasmid expressing MS2-GST fusion protein (contains MS2 binding protein which recognizes MS2 RNA, GST tag which recognizes glutathione-SH and a cytoplasmic localization signal) in HEK-293T cells. After 48 hours, cells were lysed and the complexes were affinity-purified by using GSH magnetic beads, followed by mass spectrometry. B) The immunoprecipitated protein sample was

subject to SDS-PAGE and mass spectrometry analysis to identify proteins interacting with circARHGAP35. C) RIP assay using anti-EIF3I antibody. The percentage of RIP-enriched circARHGAP35 relative to the input value was calculated by qRT-PCR. Nonspecific IgG was used as an isotype negative control. Statistical analysis was performed using unpaired Student's t tests. *P < 0.05. D) Endogenous circARHGAP35 protein was identified by mass spectrometry analysis after immunoprecipitation assay. The amino acid sequences (red letters) represent the peptides of circARHGAP35 protein. E) Representative polysome profile prepared from HCT-116 cell cytoplasmic extracts using a linear 15% to 50% sucrose gradient. The polysomes of HCT-116 cell cytoplasmic extracts were fractionated using sucrose density gradient centrifugation. Absorbance at 254 nm was measured. F) Representative polysome profile after EDTA treatment. G) The distribution of circARHGAP35, linear ARHGAP35, circPMS1 and Actin mRNA in polysomes (Fraction 6-15) and non-polysomes (Fraction 1-5). H) The m^6A RNA immunoprecipitation sequencing (meRIP-seq) data around the circARGHGAP35 loci adjacent to the translation start site. A significant peak and three RRACH fragments (R = G or A; H = A, C or U) are present at the start codon. Data were represented as mean ± SEM. Results were performed at least three independent experiments.

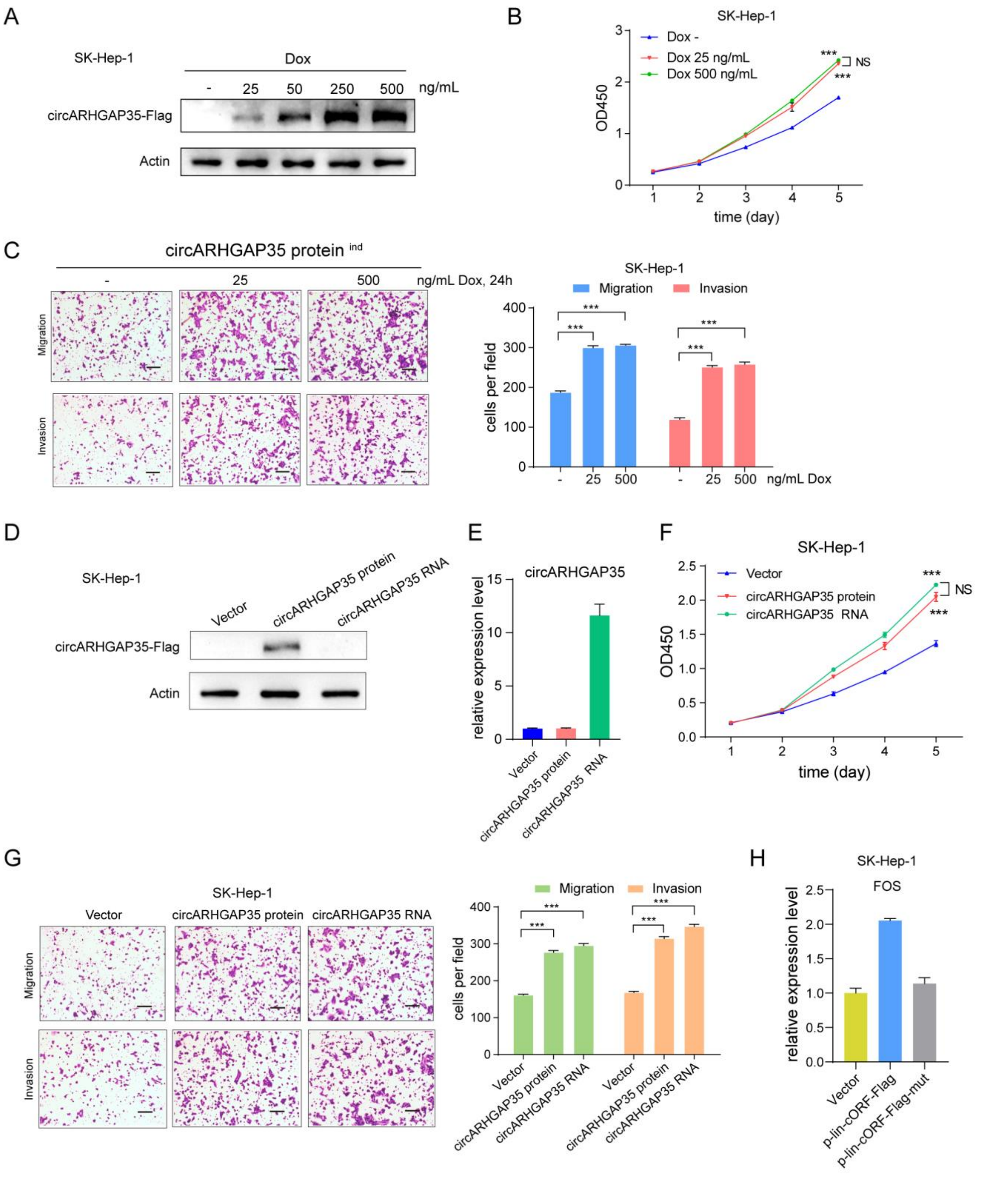

Figure S5. circARHGAP35 encodes a functional protein. A) circARHGAP35 protein was detected by western blot analysis after treatment with different concentrations of Doxycycline (Dox) in inducible SK-Hep-1 cells which were infected with pTRIPZ-circARHGAP35-ORF lentivirus. '-' means no Dox treatment. B, C) CCK-8 proliferation assays (B), and transwell migration and invasion assays (C) of inducible SK-Hep-1 cells after treatment with indicated Dox concentrations. Scale bars, 10 μm. D) Western blot validation of circARHGAP35 protein following stable overexpression of circARHGAP35 protein or RNA. circARHGAP35 protein was overexpressed by Dox treatment (25ng/mL) in inducible SK-Hep-1 cells.

circARHGAP35 RNA was overexpressed by infected with p-circARHGAP35 lentivirus. E) The expression of circARHGAP35 RNA was validated by qRT-PCR. F, G) CCK-8 proliferation assays (F), and transwell migration and invasion assays (G) after overexpression of circARHGAP35 protein or RNA. Scale bars, 10 μm. H) qRT-PCR analysis of FOS mRNA level in SK-Hep-1 cells infected with circARHGAP35 protein vector lentivirus (p-lin-cORF-Flag), mutant vector lentivirus (p-lin-cORF-Flag-mut) or vector control lentivirus. Data were represented as mean ± SEM. Results were performed at least three independent experiments; two-way ANOVA and Tukey post hoc test were performed for (B) and (F); one-way ANOVA and Dunnett post hoc test were performed for (C), (G) . *P < 0.05; **P < 0.01; ***P < 0.001.

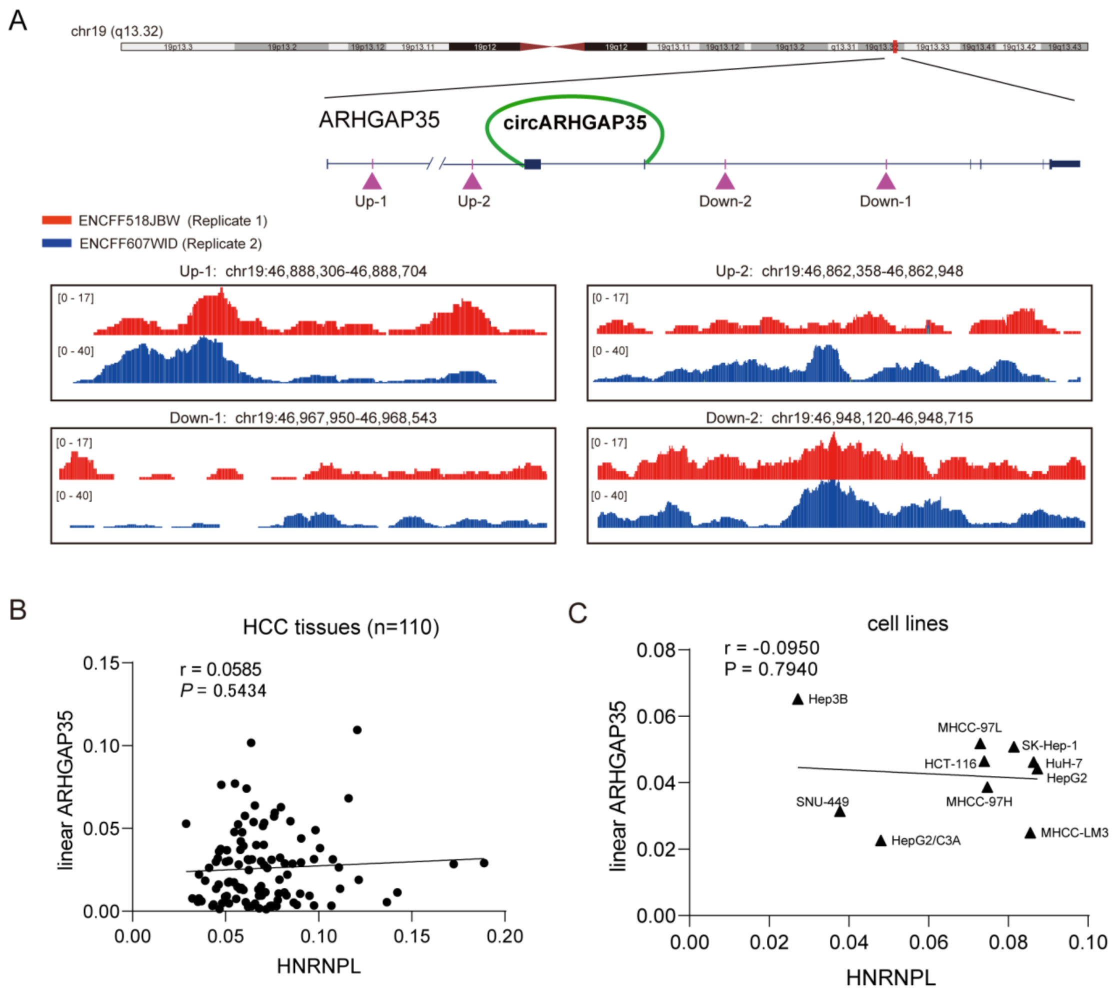

Figure S6. HNRNPL regulates circARHGAP35 formation. A) HNRNPL binding sites flanking the circARHGAP35 locus. B) Correlation between linear ARHGAP35 and HNRNPL in 110 HCC samples was determined by qRT-PCR with β-actin serving as an internal control. Statistical analysis was performed with Pearson's correlation analysis.C) Correlation between linear ARHGAP35 and HNRNPL in cancer cell lines. Statistical analysis was performed with Pearson's correlation analysis.

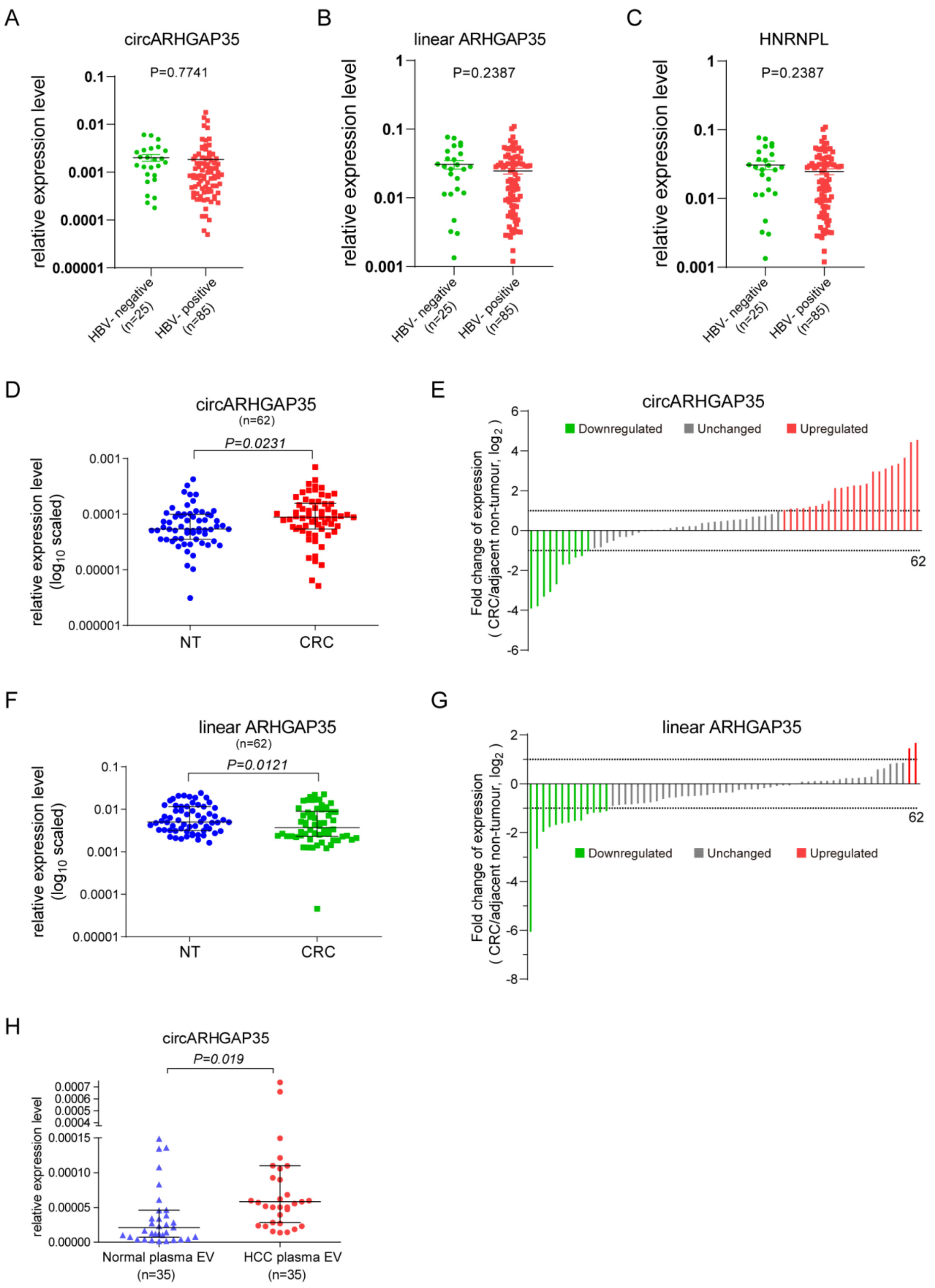

Figure S7. The expression of circARHGAP35 and linear ARHGAP35 in cancer patient samples. A-C) The expression of circARHGAP35(A), linear ARHGAP35(B) and HNRNPL(C) in HBV-positive or HBV-negative HCC samples. Data were analyzed by unpaired Student's t tests. D) The expression of circARHGAP35 in 62 paired CRC and

adjacent non-tumour (NT) cancer tissues. Data were analyzed by paired Student's t-test, n=62. E) The fold change of circARHGAP35 expression in 62 paired HCC samples (downregulated, green; unchanged, gray; upregulated, red). F) The expression of linear ARHGAP35 in 62 paired CRC and adjacent non-tumour (NT) cancer tissues. Data were analyzed by paired Student's t-test, n=62. G) The fold change of linear ARHGAP35 expression in 62 paired HCC samples (downregulated, green; unchanged, gray; upregulated, red). H) qRT-PCR analysis of circARHGAP35 levels in plasma EVs of HCC patients and healthy people (n = 35, respectively). Data were analyzed by unpaired Student's t tests.

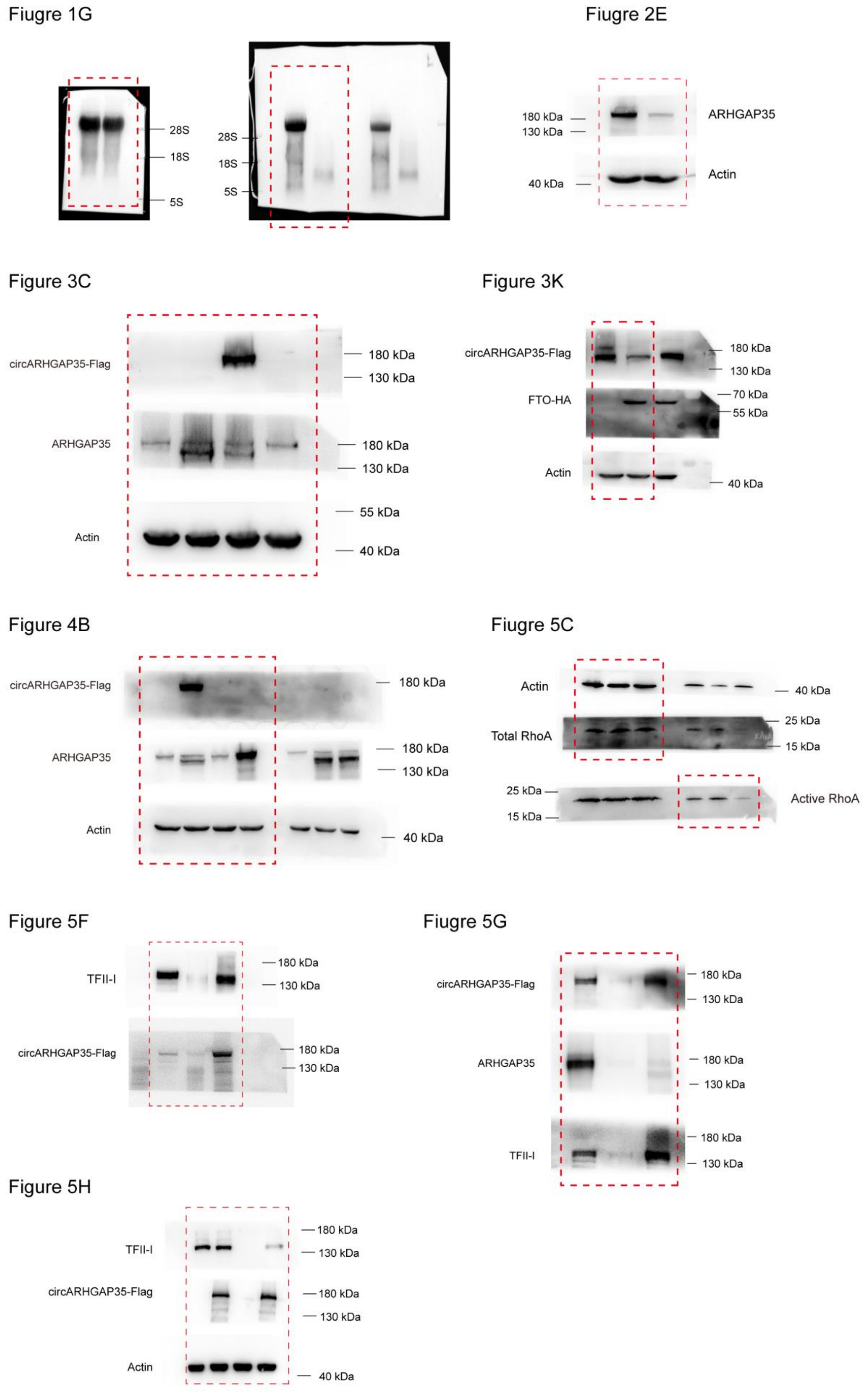

Figure S8. Uncropped blots of related figures.

Table S1. Correlation of the Clinicopathological Features With circARHGAP35 Expression in HCC

	n	circARHGAP35 expression High (＞Median)	circARHGAP35 expression Low (＜Median)	χ^2	P Value
**Gender**					
Male	92	46	46	0	1
Female	18	9	9		
**Liver cirrhosis**					
yes	87	45	42	1.831	0.4
no	22	9	13		
unknown	1	1	0		
**Family history of liver cancer**					
yes	8	4	4	0	1
no	102	51	51		
**HBsAg**					
Positive	87	39	48	4.453	0.035*
Negative	23	16	7		
**Tumor embolus**					
yes	48	25	23	0.148	0.701
no	62	30	32		
**Tumor capsule**					
yes	57	29	28	0.036	0.849
no	53	26	27		
**Tumor size (cm)**					
＞5	56	34	22	5.238	0.022*
≤5	54	21	33		
**Tumor No.**					
1	92	43	49	2.391	0.122
＞1	18	12	6		
**Serum AFP (ng/ml)**					
≤20	35	13	22	3.394	0.065
＞20	75	42	33		
**Age (years)**					
≤50	55	25	30	0.909	0.34
＞50	55	30	25		
**GGT**					
≤54 U/L	39	16	23	1.947	0.163
＞54 U/L	71	39	32		
**TNM stage**					
I + II	83	40	43	0.715	0.398
III + IV	26	15	11		
**BCLC stage**					
0 + A	18	7	11	1.063	0.303
B+C+D	92	48	44		

*, $\chi 2$ test, $P<0.05$.

Table S2. Correlation of the Clinicopathological Features With linear ARHGAP35 Expression in HCC

	n	ARHGAP35 expression		χ^2	P Value
		High (＞Median)	Low (＜Median)		
**Gender**					
Male	92	43	49	2.391	0.122
Female	18	12	6		
**Liver cirrhosis**					
yes	87	45	42		
no	22	10	12	1.285	0.526
unknown	1	0	1		
**Family history of liver cancer**					
yes	8	5	3	0.539	0.463
no	102	50	52		
**HBsAg**					
Positive	87	41	46	1.374	0.241
Negative	23	14	9		
**Tumor embolus**					
yes	48	21	27	1.331	0.249
no	62	34	28		
**Tumor capsule**					
yes	57	30	27	0.328	0.567
no	53	25	28		
**Tumor size (cm)**					
＞5	56	29	27	1.146	0.703
≤5	54	26	28		
**Tumor No.**					
1	92	48	44	1.063	0.303
＞1	18	7	11		
**Serum AFP (ng/ml)**					
≤20	35	17	18	0.042	0.838
＞20	75	38	37		
**Age (years)**					
≤50	55	24	31	1.782	0.182
＞50	55	31	24		
**GGT**					
≤54 U/L	39	25	14	4.807	0.028*
＞54 U/L	71	30	41		
**TNM stage**					
I + II	83	44	39	1.677	0.195
III + IV	26	10	16		
**BCLC stage**					
0 + A	18	9	9	0	1
B+C+D	92	46	46		

*, χ2 test, P<0.05.

Table S3. Primers and RNA sequences used in this study

		Forward primer(5'-3')	Reverse primer(5'-3')
**43 candidate circular RNAs and corresponding linear mRNAs**			
FGA	Circular	GTTGCAGTCCCTCCAGTC	AAGCTGGAACTCTGGGAG
	Linear	TGTCAGAGACTGTGATGATGTCC	TACTGGATCCCGGTAGCTTG
MARK4	Circular	AGTTGAAGCCATACACA	AGGATGCTCTCACAGTC
	Linear	GAAGGAAGCTCGAGCCAAGT	GTTCTCAGCCTTCAGGTCCC
GSE1	Circular	CCAAACAGGCGGAGGAG	GTCGCGGTGGAAAGCAT
	Linear	GCAGCCCCGGGTGAGATAAG	CAGCTCCACCTCGGCTGATA
SLC41A2	Circular	GCAGATGAATCAGAACC	TGTAGAAGTGACCCCAA
	Linear	GCCAAACATCCAGCCACAAG	CTAGCCTGAATGGCCACCAA
NNT	Circular	AAAGCTCAATACCCCAT	AGCCTCCAAGAATCACA
	Linear	GGGAGAAGCAAATGTGTCCCT	TTTGGGGACTCCAACAGTCA
UGP2	Circular	AAGACCTGGATGGATTTC	ACATTGCTTTGCTAAGAT
	Linear	GCTCAAGGTGTGCATGTGTG	GCTTTGCTAAGATCTTCCTCTTCC
SMC3	Circular	CGAGACCCAGCAAAGGAAA	CCCCCAGTTAGAGCACCCC
	Linear	CCAACACAGGGTTTCAGTGT	TAGCCTCCAGCACTTTGTCAG
SMARCA5	Circular	ATGGATACAGAGTCAAGTG	ATCTTCATCAGTGATCTCA
	Linear	CCAACACAGGGTTTCAGTGT	TAGCCTCCAGCACTTTGTCAG
REPS1	Circular	GATCTGCAGCTAAAGAGTT	GAGGTGGAGGTGGGGAATG
	Linear	GCAGGGGGCAAGTGAAAAAG	ATGCAGGTTCCTGTGCATCT
ZKSCAN1	Circular	CGCTTCAGGCGCTTCTGT	TTGCGAGCATCTCAGGTC
	Linear	TCCCAGGACATAAAGGCTGC	CCTGTAGGGTGGAATCCTGC
TMEM56	Circular	GGAAAGTGATTGGTGAC	AGCAGTTTTGTGTTGAT
	Linear	CTGAACCCAGCACCATCAGT	CAACCAGAGTCGCCAGTCAT
KDM8	Circular	AATGGTCCCAGACCCTC	CTGCTCCCTGAAATGCT
	Linear	CCAAACGCAACGAGTCTTCG	CTTTCTCCCCGAGGTCCAAC
PPP6R2	Circular	GGTGAGCCTCATCACACAG	GCAAAACAGAAAAACATTT
	Linear	AGTGTTTGATGAGCCAGCGA	TGACTCGGAGCAGCAGAAAG
MASP2	Circular	ACTCCCAGTCTGTGAGCC	TCCAGGACCTGTGATGTA
	Linear	AGGTCACTTGCCCCTGAAAT	CACAGTCAACAATGCTGCACG
VEZT	Circular	CTTTTACTAACCTCGTGAG	GTATTTTGGTAGGACTCTT
	Linear	GCACAACAGAGGGACAA	AAGCATAACGAGCAAGC
ANKIB1	Circular	CTTCAGGCTCCTCTCTTTA	ATTTGGTGGTTGTATTTCC
	Linear	GAGCTCATGACTGGGACAGG	TGGTGGTGGAGTTGGCATTT
AFTPH-F	Circular	TCGAATGATGTCTGGCTCC	CCAAAACGCACAGTGTACC
	Linear	CAGTTCCGCGTCGGAATAGA	TTTACACCTGGCTCCTTCGG
CPSF6	Circular	AGTCGACGTCATAAATCCC	CCACATCATCACCAACAGT
	Linear	TGATGCCAGTGCTGGTGATT	TCACGTCTTGATCCAGAACCA
FBXL5	Circular	GGAAATCGTTGTTGTTGGA	CCTTTGCGTATTGTGGTCA
	Linear	GCCCTTTCCTGAAGAAGTGGA	GAGCACGGAAATCGTTGTTGT
FGGY	Circular	AGAGAGATTTGCTGGGATA	GGTTGACTGGTAATGGGTG
	Linear	ACTAAAGGGCATGGTCACCG	AAGGGGATTCTTGCTGAGGC
KLHL8	Circular	CAACAGCACCAGCAAATAA	AAAGGGTAGAGAGAAGGCA
	Linear	GCCATTGTTGCCGGTTGATT	ACACAAAACAGCACACCAGC
FOCAD	Circular	TTCTCTCTCGCTTGTGTGA	AGGCTGTGCTTTTTTTGTA
	Linear	TTCCATCCACACAGATTAGCAGTT	TGCAACACTTCTCCCACAGC
PTGR1	Circular	AGAAATGGAGTGCGTTG	AGGACTTGCTGAAATGG
	Linear	AAGGAGATGCCCGCCAAAAA	TTCCCTTCATTTTTCTGCTTTGAA
DEK	Circular	AAAAGTTGAAGAAACCCCC	TTGTGCAATTGTAAATGGC
	Linear	TCTCTTCCTTACAGAGAGAGCCA	AAGCCACTGAACTGACCCAC
GDI2	Circular	AAAGCCCATACCTTTATCC	AGTCTCTTCCTCTCCCCAT
	Linear	TCGCCAGCTCCATTTTCCTC	AGATGCACTCTCTCCTCCGT
PI4KB	Circular	CTCCCTCCAAAAGGACTCA	GGCAGGCTCCACTACTGTA
	Linear	GCAACTGCAGTCCATTTGGG	GCAAGGAGAGCTGTGACTGT
TMEM181	Circular	CAGTGATAGTGGGATTTGA	GCTTGGAGAGCGTGTAGAG
	Linear	CCTGGCCAAAGGGAAGTTGT	CGGACAGGTCCACAACTGAA
ARHGAP35	Circular	AAACCAAAGCCCAAACCCC	ACCACCTCTCATCGCCAAA
	Linear	GTCAGCGGGAACAAGTCTGA	GCTCCCGGTCGTTGATTTTG
FANCL	Circular	GTTCTACTCAAGCCTTATT	TCTTCAGGCAACACTATC

	Linear	GGTAAATTCTCCTCAGAGCTCCTT	CTGGCTCAAGTACCCAGGTC
LIFR	Circular	TATTGTCCACCATCCAGG	ACCGCTCAAATGTTATCT
	Linear	GTTGCTGGGACTGAATACCCT	AGCCATTCAATCACGTGTGC
SOX5	Circular	CTTGTCTTTCCAGTCCTT	ACAAAATCAATTTGCTCC
	Linear	GGAGACGATCATAGGTGGCT	AGCCCGTCACTCTCCTCTTC
CNN2	Circular	CTGTTTGAGAGTGGGAA	GAGCGGTTGATCTTGGG
	Linear	ATCAACCGCTCCATGCAGAA	CGAGAAGAGACACCTGCACC
RBM23	Circular	ATCAGATGCCATCCTGTC	TTATCCTAGCAATACCACC
	Linear	GAAGGTTCTGGAAGACGGCA	GTCAGATCAGTTCCGGGTCC
ARHGEF12	Circular	TAAAAGAGGAGCAGCATG	ATTCACCTTCCAAACCAA
	Linear	TTCTGTTTTCTGGGGTCTGGG	GGCTCCTCGACTCCTCAACT
RHOBTB3	Circular	TCGTTGGGAAGAATTGGA	TGTGACGCTTCAGCCTTT
	Linear	AGGCAACCCACCATTACGAG	TCCCACTGAAAAGCACCTGA
C3	Circular	TCTGGGAACTCACTTCGG	GTGGGAGATTCTGGCTGT
	Linear	CAAGCGCATTCCGATTGAGG	CCTGAGTGCAAGATGACGGT
ENPP1	Circular	GCCTCAAGTCTGGCACAT	AGCATTCATTTTGGGATC
	Linear	CAGCATTGTCACCGGATTGT	GCCAGACTTGAGGCCTTGAT
ZCCHC6	Circular	GCCCCAAATGATAGATGT	TGACAGGAATACCAAATAC
	Linear	GCGGCGAAGGTGGTGC	ACTGCCATGGCCTTTAGCAT
MED13L	Circular	AGGATTGGGAAATGGTTT	TGTGGATCGCTTTGAAGA
	Linear	GGTGAGGCGAGCCAGAAATA	CCTCCGTGACCTGTTTGGTA
HMGCS1	Circular	GACAGAATAGCAGCGGTCTA	GCGTCCCACTCCAAATGA
	Linear	GGACTGTCCTTTCGTGGCTC	GGCATGGTGAAAGAGCTGTG
PTGR1	Circular	AGCGTCTCCTGATGGTTA	AGAAATGGAGTGCGTTGT
	Linear	CAGTCCCAGCCGCCTTC	GCTTCAAGCAGGACCTCTCC
CBFA2T2	Circular	GAGTTGCTCATGGAGGTG	AAGTTTGTGGCTTCTTGG
	Linear	GCCTTCCAGCTTGGTCCTG	GGGAGGTGAGGATCTGGACT
LONP2	Circular	TTTAGTGGGCGGCTGGTA	AAGCCAAACCTATTGCTACTCC
	Linear	TGCAGTACAAATTTTAGATGCTGTG	GGGCGTATTGCTATAACCCTCT
Actin	Linear	TTGTTACAGGAAGTCCCTTGCC	ATGCTATCACCTCCCCTGTGTG
**Putative circARHGAP35 isoforms**			
circARHGAP35.2		GGAACACTAAGGAAGAAAT	CTGGACATGCAGCAAAAAT
circARHGAP35.3		CCCGTATAACATGCAGATC	CGGTATTCACCGTAAAGTC
circARHGAP35.4		TTGTACTTGTCTCAATGGG	AGTGCTGAAATCAGGTCTC
circARHGAP35.5		TTCCTCTTGTTTCCCCCGC	CTGCCACAGTCCCTGCCTG
circARHGAP35.6		TGGGTGGGAGGGCGTTGGC	AAGCGGGAGTGGGGGCAGC
**Northern blotting**			
circARHGAP35-probe		CAGGCATCCCAGGGTTAT	GATCACTAATACGACTCACTATAG GGTTCGGACATCTTGCTTTCTT
linear ARHGAP35-probe		GGCTGGTGCCATGAAGAG	GATCACTAATACGACTCACTATAG GGAGGCGTGGAAGTGAGGAA
circHIPK3-probe		CGGCAGCCTTACAGGGTTA	TAATACGACTCACTATAGGGAGAC AACTGCTTGGCTCTACTT
**gRNA**			
gRNA targeting ARHGAP35		CACCGTATAACATGCAGATCGACT	AAACAGTCGATCTGCATGTTATAC
**shRNA**			
shRNA targeting circARHGAP35		CACCGTGAAGCCACAGGAAGAAATTC AAGATTTCTTCCTGTGGCTTCATTTTTT	AAACAAAAAATGAAGCCACAGGA AGAAATCTTGAATTTCTTCCTGTGG CTTCAC
**Constructs**			
p-circARHGAP35		CGGAATTCTGAAATATGCTATCTTACA GGAAGAAATGTTGGCTATTTG	CGGGATCCTCAAGAAAAAATATAT TCACCTGTGGCTTCAATGTACTCA
p-lin-cORF-Flag		AGAAGATTCTAGAGCTAGCGATGATG GACTACAAAGACCATGACGGTGATTA TAAGGATCATGACATCGATTACA	GCAGATCCTTCGCGGCCGCGTCAC TGTTCACCACCTCTCATCGCCAAAT AGCCAACATTTCTTCCTGTGGCTTC AATGTACTCAA

p-lin-FL	AGAAGATTCTAGAGCTAGCGATGATG ATGGCAAGAAAGC	GCAGATCCTTCGCGGCCGCGTCAC AGCGTGTGTTCGGCTT
pTRIPZ-circARHGAP35-ORF	GTGAACCGTCAGATCGCAATGATGGA CTACAAAGACCA	CTAAAGTAGCCCCTTGAATTTCACT GTTCACCACCTCTCA
**HNRNPL related**		
Up-1	GGAGACAAACTCAGCACCAT	CCCTGGGACCTTGAAATA
Up-2	CCCACGTTACGTCGCTCCTT	CGGGACCTGACCAGCCAAT
Down-1	TTGGGCCTGCCGTGGAAAGGA	ATCACACACGCCACCACCAC
Down-2	TCAGAAGGGCTGAGACAG	TTTCAAACAGTAAACCACC
U2	ATCGCTTCTCGGCCTTTTGG	AGGTCGATGCGTGGAGTGGA
HNRNPL mRNA	ATGAGCTGGGAGTGAAGC	TCAGGAAGCCCAGAGTCT
**Others**		
circGFP	GCCGGACACGCTGAACTT	TCTCGGCATGGACGAGCT
circPMS1	CTTGAAAGGAGTCGAACTGTTGCC	GATCTCCTCATGAGCTTTGGTATCC

Table S4. Antibodies used in this study

ANTIBODIES	SOURCE	IDENTIFIER
p190-A RhoGAP (D8Q6C) (ARHGAP35)	Cell Signaling Technology	Cat. #12164
TFII-I	Cell Signaling Technology	Cat. #4562
HA	Cell Signaling Technology	Cat. #3725
FLAG	Sigma	Cat. F1804
β-actin	Proteintech	Cat. 60008-1-IG
HNRNPL	Santa Cruz Biotechnology	Cat. sc-32317

Table S5. siRNA sequences used in this study

siRNA	Sequences
circARHGAP35 siRNA	GCCACAGGAAGAAATGTTG
both circular/linear ARHGAP35 siRNA	GCACCTTAGTGCAACTCAT
linear ARHGAP35 siRNA	GCACACATTTACTCTGCAT
TFII-I siRNA-1	CGAGAACTATGATCTTGCA
TFII-I siRNA-2	GTCGTGTGATGGTAACAGA
TFII-I siRNA-3	GTATGAAACAGACGTGTTT
**siRNAs for 63 RBPs**	
CSTF2 siRNA-1	GGTTGGACCTGTTGTTAGT
CSTF2 siRNA-2	CTGAATGGGCGCGAATTCA
CSTF2 siRNA-3	AAGAGGACCCATGCTAGAT
HNRNPA2B1 siRNA-1	GGAGAGTAGTTGAGCCAAA
HNRNPA2B1 siRNA-2	GCTACGGAGGTGGTTATGA
HNRNPA2B1 siRNA-3	AGCTGTTTGTTGGCGGAAT
HNRNPF siRNA-1	GGAAGTTAGGTCATACTCA
HNRNPF siRNA-2	ACCGGTACATTGAGGTGTT
HNRNPF siRNA-3	AAGCGACCGAGAACGACAT
DHX8 siRNA-1	GCAGTGGGTTCTTCCGTAA
DHX8 siRNA-2	GGAGTTTGGTTGTTGCTTA
DHX8 siRNA-3	GCACTGAGCTGGACAATCA
HNRNPH1 siRNA-1	GGTCCAAATAGTCCTGACA
HNRNPH1 siRNA-2	GATCCACCACGAAAGCTTA
HNRNPH1 siRNA-3	GTTCGCAACTCATGAAGAT
CLK2 siRNA-1	GGAGATGCCTACTATGACA
CLK2 siRNA-2	CTATCGGCATTCCTATGAA

CLK2 siRNA-3	GAGAGCATCTAGCCATGAT
GEMIN2 siRNA-1	GATCGAAGCAGCTCAATGT
GEMIN2 siRNA-2	GTAGAGCCTTGCGACTTGA
GEMIN2 siRNA-3	AAAGTCCTGGAATAGATTA
EFTUD2 siRNA-1	GATGGAGCCTTACTACTTT
EFTUD2 siRNA-2	GAGCAGACATTACCTGTTA
EFTUD2 siRNA-3	CAAGGCTCTTCTTGGTTCA
EEF1E1 siRNA-1	GCAACATCTGTCTAGTGTT
EEF1E1 siRNA-2	ACCTGACAGTTCAAGAAAA
EEF1E1 siRNA-3	CACACAGAGGTAGGGAACT
HNRNPL siRNA-1	GACGGGTCTTGCAGTTACA
HNRNPL siRNA-2	GTCAGTCATACGGGTTGGA
HNRNPL siRNA-3	GTCCATACCCTTACACTCT
HNRNPH3 siRNA-1	GGCTACGGAAGAGATGGAA
HNRNPH3 siRNA-2	ATGGGATAACATTGACGAT
HNRNPH3 siRNA-3	GACCATATGATAGACCAAT
HNRNPK siRNA-1	GGATCCGAAGATCGGATCA
HNRNPK siRNA-2	ATCCCAATTTTTACGATGA
HNRNPK siRNA-3	AAGGTGGTCAGCGGATTAA
CPSF1 siRNA-1	TGATCAGCGTCAAGAACTT
CPSF1 siRNA-2	CCCACTATGTGGCTTACCA
CPSF1 siRNA-3	ACCGCAACCTCATGGTGTA
DAZAP1 siRNA-1	TCACGGAGGTAGTCATGAT
DAZAP1 siRNA-2	ACACGCTAGATGGCCGAAA
DAZAP1 siRNA-3	AAAGGACCCAGGAGCGATA
DHX35 siRNA-1	GCATGAGAATTGCAGAGTT
DHX35 siRNA-2	GCAGCGAGGTTTCATTCTA
DHX35 siRNA-3	CAAAGACTGTCGCCTAACT
ALYREF siRNA-1	GCACGATCTTTTCGACAGT
ALYREF siRNA-2	GAGGTGGCATGACTAGAAA
ALYREF siRNA-3	ACGACATCATTAAACTGAA
CPSF3 siRNA-1	GGACCCATATCCATGAGAA
CPSF3 siRNA-2	GTAACACTGTCCACGATAT
CPSF3 siRNA-3	GCAGTGACCTTAAACTTCA
POLR2G siRNA-1	CCACCATGTTACAAGACAA
POLR2G siRNA-2	GCTCCCTGATGGACGATTA
POLR2G siRNA-3	GCCGAGGCTTTGTCCTTTA
PRPF3 siRNA-1	CTGGGAGTATATCTTACCA
PRPF3 siRNA-2	GGAGTAAAGAAGCGACGAA
PRPF3 siRNA-3	GGGAACAATTCAAGCAACA
KIF2A siRNA-1	TGGTGATGTTCGTCCAATA
KIF2A siRNA-2	TTCCGTGCAAGTAAACTCA
KIF2A siRNA-3	TAAGAATGACCCTCCTTCA
LAMB2 siRNA-1	CAAGGATGATCGCATCCAA
LAMB2 siRNA-2	GTGCAACCCTCAAGGTTCA
LAMB2 siRNA-3	CCTACAAGCTGCATCTGAA
HNRNPM siRNA-1	GGTGTGGCGTGGTTAAGTT
HNRNPM siRNA-2	GAGAGATTGACGTTCGAAT
HNRNPM siRNA-3	GAAGGCCTGCCAGATATTT
NONO siRNA-1	GAAGGCTCCTTCCTGCTAA
NONO siRNA-2	CAGGCGAAGTCTTCATTCA
NONO siRNA-3	AGGTCATGCTAATGAGACA
PPARGC1A siRNA-1	CACCACTCCTCCTCATAAA
PPARGC1A siRNA-2	CAGAGACACTAGACAGTCT
PPARGC1A siRNA-3	GGCACGCAATCCTATTCAT

PRPF6 siRNA-1	GAAGCGGGTTCTTCGGAAA
PRPF6 siRNA-2	GGATCTAAATGACACCAAT
PRPF6 siRNA-3	CTCGGAACCTTATCATGAA
PRPF40B siRNA-1	GGCAAACCTTATTACTATA
PRPF40B siRNA-2	CCACCTGGATGGAGCTATA
PRPF40B siRNA-3	GCAATATCCAGGCCCTAAA
LSM4 siRNA-1	CCGACGAGATCATCGACAT
LSM4 siRNA-2	ACAACTGGATGAACATTAA
LSM4 siRNA-3	GCAGCACCATCAAGTACCT
PPIH siRNA-1	GCATGAAGATCGAGCTCTT
PPIH siRNA-2	GCAGACGTTGTGCCTAAGA
PPIH siRNA-3	GGGTTCCAATAGGATACAA
PRPF31 siRNA-1	GCTACGAACTGAAGGATGA
PRPF31 siRNA-2	GCCAAGCTATGGGATAGTA
PRPF31 siRNA-3	CAAGCAAGCCAAAGCTTCA
HNRNPR siRNA-1	AGGTAGAGCTGGCTATTCA
HNRNPR siRNA-2	ACTGTCCGGTCAGAATAGA
HNRNPR siRNA-3	AGGGTTTGGTGGACGTTAT
LSM2 siRNA-1	TCTCAACATCAAACTAACT
LSM2 siRNA-2	GTGTCACAGACCCTGAGAA
LSM2 siRNA-3	GTGGAACCCTCCATTCTGT
PHF5A siRNA-1	AGAGATGGCTGCCCAAAGA
PHF5A siRNA-2	GCGCATATGTGATGAGTGT
PHF5A siRNA-3	GGTCTCTGATGCCTATTAT
HNRNPLL siRNA-1	GAACACCCTTCTTCGTTTA
HNRNPLL siRNA-2	CGTGTACATTGCTGGTCAA
HNRNPLL siRNA-3	GTACCAGCAGCTACAAAGA
LSM7 siRNA-1	CGATCCGGGTAAAGTTCCA
LSM7 siRNA-2	ACGTCCGTGGTGCTAATCT
LSM7 siRNA-3	ACCCACTCCTCAACCTTGT
SERPINC1 siRNA-1	CCAACAGGGTGACTTTCAA
SERPINC1 siRNA-2	GAGGACGGCTTCAGTTTGA
SERPINC1 siRNA-3	GAGTCGTGTTCAGCATCTA
SNRPA siRNA-1	CAGATATCATTGCCAAGAT
SNRPA siRNA-2	TCGTGGAGTTTGACAATGA
SNRPA siRNA-3	CTGAGAATCCACCGAATCA
PTEN siRNA-1	GGAATATCTAGTACTTACT
PTEN siRNA-2	GGTGTAATGATATGTGCAT
PTEN siRNA-3	ACCGCCAAATTTAATTGCA
SNRPB siRNA-1	TGGTCTCAATGACAGTAGA
SNRPB siRNA-2	CTGGTATGAGACCTCCTAT
SNRPB siRNA-3	GCCAAAGAACTCCAAACAA
SATB1 siRNA-1	GGTACGCGATGAACTGAAA
SATB1 siRNA-2	GTCCTCAATTGGTCAACCA
SATB1 siRNA-3	GGGCTCGTATCAACACCTA
SFPQ siRNA-1	CCAGAAATTTCCACCTCTA
SFPQ siRNA-2	GGATGATCGTGGAAGATCT
SFPQ siRNA-3	GGAGGAACGACGTAGAAGA
SF3B4 siRNA-1	GGAAGCCAATACGGGTGAA
SF3B4 siRNA-2	GTCCTATCACCGTATCTTA
SF3B4 siRNA-3	GCATCAGCTCACAACAAAA
SF3B2 siRNA-1	GGACGTGTTTGGAACCAAT
SF3B2 siRNA-2	GAGACACCTCAGCTCTTCA
SF3B2 siRNA-3	GGAAGCGTAGGAACCGAAA
SRSF11 siRNA-1	GGATACCTCTAGTAAAGAA

SRSF11 siRNA-2	GAGCAAACTTGAACTCTCA
SRSF11 siRNA-3	GGCACAAGATCTCCTAAAA
SRSF10 siRNA-1	GACGGCAGATTGAAATACA
SRSF10 siRNA-2	GTCGGTCTTTTGATTACAA
SRSF10 siRNA-3	GACAGATACAGACGTTCTA
SNRPC siRNA-1	CTCAGAGCCTGATTGACAA
SNRPC siRNA-2	GCTGCATTTCAACAAGGAA
SNRPC siRNA-3	CTCCCATGATGCCAATGAT
SNRPD2 siRNA-1	GCCGCAACAATAAGAAACT
SNRPD2 siRNA-2	GACTGAGGTACCCAAGAGT
SNRPD2 siRNA-3	GCGAGAGGAGGAGGAATTT
RBMXL1 siRNA-1	TGAGAAAGCTCTTGAAACA
RBMXL1 siRNA-2	GATGGTGGATATTCCATGA
RBMXL1 siRNA-3	GGAGGAAGAGCTCCTCTAT
PTBP2 siRNA-1	GTGTTACTCTGTCTAAACA
PTBP2 siRNA-2	GCAGAAGAGGATCTACGAA
PTBP2 siRNA-3	GAGAGGGACTTGATGATCA
RALY siRNA-1	AGACCATCTTCTCTAAGTA
RALY siRNA-2	GCAGCATCTGCCATATACA
RALY siRNA-3	GAGTCTTCATTGGAAACCT
SRSF2 siRNA-1	CACGAAGGTCCAAGTCCAA
SRSF2 siRNA-2	GTGTCCAAGAGGGAATCCA
SRSF2 siRNA-3	TCGGTCTCCAGATCTCGTT
SRSF4 siRNA-1	GAATCACGCTCCAGATCAA
SRSF4 siRNA-2	GACTTATTGTGGAGAATTT
SRSF4 siRNA-3	CGATCTCGGTCCAAGTCTA
SRSF6 siRNA-1	GACCACCTGTTCGTACAGA
SRSF6 siRNA-2	AGGCGATCTTACTCTGGAA
SRSF6 siRNA-3	GCAGATCTAAGGATGAGTA
SRSF7 siRNA-1	GGGACATTATGCTTATGAT
SRSF7 siRNA-2	CCTCGACGATCAAGATCTA
SRSF7 siRNA-3	CGAAGATCCTAGAGATGCA
TRA2B siRNA-1	GGACTACTATAGCAGATCA
TRA2B siRNA-2	GGCGTAGGATCAGAGTTGA
TRA2B siRNA-3	CGGTCACCTTCTCCTTACT
SRSF3 siRNA-1	CTATGTGGCTGCCGTGTAA
SRSF3 siRNA-2	CGAGATGATTATCGTAGGA
SRSF3 siRNA-3	CGATCTAGGTCAAATGAAA
THOC2 siRNA-1	GGGCAAGATTTATCTGGAA
THOC2 siRNA-2	GGACAAGCTTCTTGAAGAA
THOC2 siRNA-3	GCCGCAAAATTGATACTCA
USP39 siRNA-1	TCAAGAGATTCACTAAGAA
USP39 siRNA-2	GCAGTTGTACTTTGCAGTA
USP39 siRNA-3	TCCCGGGCATATGATGGTA
SUGP2 siRNA-1	CACCCAGAGTTGATAATGA
SUGP2 siRNA-2	GCACTAACCAAATCCAGAA
SUGP2 siRNA-3	GCTTTGACATCATAGATAA
TUBG1 siRNA-1	GGTCCAGCCTTACAATTCA
TUBG1 siRNA-2	GACGCAGAATGCAGACTGT
TUBG1 siRNA-3	GAACCTGTCGCCAGTATGA
THOC5 siRNA-1	GTGCAGGAGTACCTGTTTA
THOC5 siRNA-2	GGGTTCAGTTGGACGACAA
THOC5 siRNA-3	GAGAGTGCCTATCTAACAA
SRSF9 siRNA-1	GGTCGAGTATCTCAGAAAA
SRSF9 siRNA-2	ACACCAAATTCCGCTCTCA

SRSF9 siRNA-3	GGAATATGCCCTGCGTAAA
SUGP1 siRNA-1	GTAGCTTCTTGCAGCAGTT
SUGP1 siRNA-2	GCAGGGAATTCCTCTACTA
SUGP1 siRNA-3	GAGCCTGACTACTCAGAGT
UPF3B siRNA-1	GATGCAGCTTTACCAACCA
UPF3B siRNA-2	TCAGGAATATCCCGCTATA
UPF3B siRNA-3	GCGCATACTTCGAGAAAGA

Table S6. Sequences of circARHGAP35 RNA and protein

circARHGAP35 RNA:

GAAGAAATGTTGGCTATTTGGCGATGAGAGGTGGTGAACAGTGACCATACTGGTATACAACACTATGGGACCTGGCATTTTTGCTGC
ATGTCCAGCCCACCCCCACTAATAATGTAGGAAGCTGTCTGGTCCATTGGAAACACTAATCTGATCTCAGAAGTGGCTGATCGTGGC
AGGATGTGTCGACGATGATGATGGCAAGAAAGCAAGATGTCCGAATTCCCACCTACAACATCAGTGTGGTGGGATTATCTGGGACCG
AGAAGGAAAAGGGCCAGTGTGGGATTGGAAAGTCTTGTTTGTGCAACCGCTTCGTGCGCCCGAGTGCTGACGAGTTTCACTTGGACC
ATACCTCCGTCCTCAGCACCAGTGACTTTGGAGGGCGAGTGGTCAATAATGACCACTTTCTCTACTGGGGAGAAGTTAGCCGCTCCCT
GGAGGATTGTGTGGAATGTAAGATGCACATTGTGGAGCAGACTGAATTTATTGATGATCAGACTTTTCAACCTCATCGAAGCACGGC
CCTGCAGCCCTATATCAAGAGAGCTGCTGCGACCAAGCTTGCATCAGCTGAAAAACTCATGTACTTTTGCACTGACCAGCTGGGGCT
GGAGCAGGACTTTGAGCAGAAACAAATGCCAGACGGAAAGCTGCTGGTTGATGGTTTTCTTCTTGGTATTGATGTTAGCAGGGGCAT
GAATAGGAACTTTGATGACCAGCTCAAGTTTGTCTCCAATCTCTACAATCAGCTTGCAAAAACAAAAAAGCCCATAGTGGTGGTCCT
GACTAAGTGTGACGAAGGTGTTGAGCGGTACATTAGAGATGCACATACTTTTGCCTTAAGCAAAAAGAACCTCCAGGTTGTGGAGAC
CTCAGCGAGATCCAATGTAAACGTGGACTTGGCTTTCAGCACCTTAGTGCAACTCATTGATAAAAGTCGGGGAAAGACAAAAATCAT
TCCTTATTTTGAAGCTCTCAAGCAGCAGAGTCAGCAGATAGCTACAGCAAAAGACAAGTATGAGTGGCTGGTGAGTCGCATTGTGAA
AAACCACAATGAGAACTGGCTGAGTGTCAGCCGAAAGATGCAGGCCTCTCCAGAATACCAGGACTATGTCTACCTGGAAGGGACTC
AGAAAGCCAAGAAGCTGTTTCTACAGCACATCCACCGCCTCAAGCATGAGCATATCGAGCGTAGGAGAAAGCTGTACCTGGCAGCC
CTGCCATTAGCTTTTGAAGCTCTTATACCTAATCTAGATGAAATAGACCACCTAAGCTGCATAAAAGCCAAAAAGCTCTTAGAAACC
AAGCCAGAATTCTTGAAGTGGTTTGTTGTGCTTGAAGAGACCCCATGGGATGCCACCAGTCACATTGACAACATGGAAAACGAACGG
ATTCCCTTTGATTTAATGGATACCGTCCCTGCAGAGCAGCTATACGAGGCCCACTTAGAGAAGCTGAGGAACGAAAGGAAAAGAGTT
GAGATGCGAAGGGCGTTTAAAGAAAACCTGGAGACTTCTCCTTTCATAACTCCCGGAAAGCCTTGGGAAGAGGCCCGTAGTTTTATT
ATGAATGAGGATTTCTACCAGTGGCTGGAGGAATCTGTATACATGGATATTTATGGCAAACACCAAAAGCAAATTATAGATAAAGCA
AAGGAAGAATTTCAGGAGTTGCTTTTGGAATATTCAGAATTGTTTTATGAACTGGAGCTGGATGCTAAGCCCAGCAAGGAGAAGATG
GGTGTTATTCAGGATGTTCTGGGAGAGGAACAGCGATTTAAAGCATTACAAAAGCTCCAAGCAGAGCGTGATGCCCTTATTCTGAAA
CACATTCATTTTGTGTACCACCCAACAAAGGAGACATGCCCCAGCTGCCCAGCTTGTGTGGACGCTAAGATTGAGCACTTGATTAGTT
CTCGGTTTATCCGGCCGTCTGACCGGAATCAGAAAAATTCACTCTCTGACCCTAACATTGATAGAATCAACTTGGTTATATTGGGCAA
AGACGGCCTTGCCCGAGAGTTGGCCAATGAGATTCGAGCTCTTTGTACAAATGATGACAAGTATGTGATAGATGGTAAAATGTATGA
GCTTTCCCTGAGGCCAATAGAGGGGAATGTCAGGCTTCCTGTGAACTCTTTCCAGACGCCAACATTTCAGCCCCACGGCTGTCTCTGC
CTTTACAATTCAAAGGAATCGCTATCCTATGTAGTGGAAAGTATAGAGAAGAGTAGAGAGTCCACGCTGGGCCGGCGGGATAATCAT
TTAGTCCATCTCCCCCTTACATTAATTTTGGTTAACAAGAGAGGAGACACCAGTGGAGAGACTCTGCATAGCTTAATACAGCAAGGT
CAACAAATTGCTAGCAAACTTCAGTGTGTCTTTCTCGACCCTGCTTCTGCTGGCATTGGTTACGGACGCAACATTAATGAAAAGCAAA
TCAGTCAAGTTTTGAAGGGACTCCTGGACTCTAAGCGTAACTTAAACCTGGTCAGTTCTACTGCTAGCATCAAAGATTTGGCTGATGT
TGATCTGCGAATTGTTATGTGTCTGATGTGTGGAGATCCTTTTAGTGCAGATGACATACTTTTTCCTGTCCTTCAGTCCCAAACCTGTA
AATCTTCCCATTGTGGAAGCAACAACTCTGTTTTACTTGAACTACCAATCGGACTGCACAAGAAGCGGATTGAACTGTCTGTTCTTTC
ATACCATTCCTCCTTTAGCATCAGAAAGAGCCGGTTGGTTCATGGGTACATTGTTTTTTATTCAGCCAAACGTAAGGCCTCTTTGGCTA
TGTTACGTGCCTTTCTTTGTGAAGTGCAGGATATTATCCCTATTCAGCTTGTAGCACTCACTGATGGCGCTGTAGATGTCCTGGACAAT
GACTTAAGTAGGGAACAGCTAACTGAGGGGGAGGAGATTGCTCAAGAAATTGACGGAAGGTTCACAAGCATCCCCTGTAGCCAACC
CCAGCATAAACTTGAGATCTTTCACCCATTTTTTAAAGATGTGGTGGAAAAAAAGAACATAATCGAGGCTACTCATATGTACGATAA
TGCTGCCGAGGCCTGTAGCACCACCGAAGAGGTGTTTAACTCCCCCCGGGCAGGATCACCGCTCTGCAACTCAAACCTGCAGGATTC
AGAAGAAGATATCGAGCCATCTTACAGCCTGTTTCGAGAAGACACATCACTGCCTTCTCTGTCCAAAGACCATTCTAAGCTCTCTATG
GAACTGGAGGGAAATGATGGGCTGTCTTTCATTATGAGCAATTTTGAGAGTAAACTGAACAACAAAGTACCTCCGCCAGTCAAACCA
AAGCCTCCTGTCCATTTTGAAATTACAAAGGGGGATCTATCTTATTTAGACCAAGGCCATAGGGATGGACAGAGGAAGTCTGTGTCT
TCTAGCCCCTGGCTGCCTCAGGATGGGTTTGATCCTTCTGACTATGCTGAACCCATGGATGCTGTGGTGAAGCCAAGGAATGAAGAA
GAAAACATATACTCCGTGCCCCATGACAGCACCCAAGGCAAAATCATCACCATTCGGAATATCAACAAAGCCCAGTCCAACGGCAG
CGGGAATGGTTCTGACAGTGAAATGGACACCAGCTCTCTAGAGCGAGGGCGCAAGGTTTCCATCGTGAGCAAGCCAGTGCTGTACAG
GACGAGATGCACCCGGCTGGGGCGGTTTGCTAGTTACCGGACCAGCTTCAGCGTGGGGAGTGATGATGAGCTGGGGCCCATCCGGA
AGAAAGAGGAGGATCAGGCATCCCAGGGTTATAAAGGGGACAATGCTGTCATTCCATACGAAACAGACGAAGACCCGCGGAGGAG
GAATATTCTTCGCAGCCTAAGGAGGAACACTAAGAAACCAAAGCCCAAACCCCGGCCATCCATCACAAAGGCAACCTGGGAGAGTA
ACTATTTTGGGGTGCCCTTAACAACTGTCGTGACTCCAGAGAAGCCGATCCCCATTTTTATTGAAAGATGTATTGAGTACATTGAAGC
CACAG

circARHGAP35 protein:

MMMARKQDVRIPTYNISVVGLSGTEKEKGQCGIGKSCLCNRFVRPSADEFHLDHTSVLSTSDFGGRVVNNDHFLYWGEVSRSLEDCVECK
MHIVEQTEFIDDQTFQPHRSTALQPYIKRAAATKLASAEKLMYFCTDQLGLEQDFEQKQMPDGKLLVDGFLLGIDVSRGMNRNFDDQLKF
VSNLYNQLAKTKKPIVVVLTKCDEGVERYIRDAHTFALSKKNLQVVETSARSNVNVDLAFSTLVQLIDKSRGKTKIIPYFEALKQQSQQIAT
AKDKYEWLVSRIVKNHNENWLSVSRKMQASPEYQDYVYLEGTQKAKKLFLQHIHRLKHEHIERRRKLYLAALPLAFEALIPNLDEIDHLS
CIKAKKLLETKPEFLKWFVVLEETPWDATSHIDNMENERIPFDLMDTVPAEQLYEAHLEKLRNERKRVEMRRAFKENLETSPFITPGKPWE
EARSFIMNEDFYQWLEESVYMDIYGKHQKQIIDKAKEEFQELLLEYSELFYELELDAKPSKEKMGVIQDVLGEEQRFKALQKLQAERDALI
LKHIHFVYHPTKETCPSCPACVDAKIEHLISSRFIRPSDRNQKNSLSDPNIDRINLVILGKDGLARELANEIRALCTNDDKYVIDGKMYELSLR
PIEGNVRLPVNSFQTPTFQPHGCLCLYNSKESLSYVVESIEKSRESTLGRRDNHLVHLPLTLILVNKRGDTSGETLHSLIQQGQQIASKLQCVF
LDPASAGIGYGRNINEKQISQVLKGLLDSKRNLNLVSSTASIKDLADVDLRIVMCLMCGDPFSADDILFPVLQSQTCKSSHCGSNNSVLLELP
IGLHKKRIELSVLSYHSSFSIRKSRLVHGYIVFYSAKRKASLAMLRAFLCEVQDIIPIQLVALTDGAVDVLDNDLSREQLTEGEEIAQEIDGRF
TSIPCSQPQHKLEIFHPFFKDVVEKKNIIEATHMYDNAAEACSTTEEVFNSPRAGSPLCNSNLQDSEEDIEPSYSLFREDTSLPSLSKDHSKLS
MELEGNDGLSFIMSNFESKLNNKVPPPVKPKPPVHFEITKGDLSYLDQGHRDGQRKSVSSSPWLPQDGFDPSDYAEPMDAVVKPRNEEENI
YSVPHDSTQGKIITIRNINKAQSNGSGNGSDSEMDTSSLERGRKVSIVSKPVLYRTRCTRLGRFASYRTSFSVGSDDELGPIRKKEEDQASQG
YKGDNAVIPYETDEDPRRRNILRSLRRNTKKPKPKPRPSITKATWESNYFGVPLTTVVTPEKPIPIFIERCIEYIEATGRNVGYLAMRGGEQ-

The start codon (ATG) and stop codon (TGA) are shown in blue letters. The red letters represent the specific sequence of circARHGAP35 protein.
